# Increasing educational attainment and mortality reduction: a systematic review and taxonomy

**DOI:** 10.1186/s12889-017-4754-1

**Published:** 2017-09-18

**Authors:** Elena Byhoff, Mary C. Hamati, Robyn Power, Sarah A. Burgard, Vineet Chopra

**Affiliations:** 1 Department of Medicine, Tufts Medical Center, Tufts University School of Medicine, 800 Washington St Box #63, Boston, MA 02111 USA; 2 University of Michigan, Ann Arbor, MI USA; 3 Department of Sociology, University of Michigan, Ann Arbor, MI USA; 4 Division of General Internal Medicine, University of Michigan, Ann Arbor, MI USA

**Keywords:** Education, Mortality, Systematic review, Taxonomy

## Abstract

**Background:**

Understanding the relationship between increasing educational attainment and mortality reduction has important policy and public health implications. This systematic review of the literature establishes a taxonomy to facilitate evaluation of the association between educational attainment and early mortality.

**Methods:**

Following PRISMA guidelines, we searched Ovid Medline, Embase, PubMed and hand searches of references for English-language primary data analyses using education as an independent variable and mortality as a dependent variable. Initial searches were undertaken in February 2015 and updated in April 2016.

**Results:**

One thousand, seven hundred and eleven unique articles were identified, 418 manuscripts were screened and 262 eligible studies were included in the review. After an iterative review process, the literature was divided into four study domains: (1) all-cause mortality (*n* = 68, 26.0%), (2) outcome-specific mortality (*n* = 89, 34.0%), (3) explanatory pathways (*n* = 51, 19.5%), and (4) trends over time (*n* = 54, 20.6%). These four domains comprise a novel taxonomy that can be implemented to better quantify the relationship between education and mortality.

**Conclusions:**

We propose an organizational taxonomy for the education-mortality literature based upon study characteristics that will allow for a more in-depth understanding of this association. Our review suggests that studies that include mediators or subgroups can explain part, but not all, of the relationship between education and early mortality.

**Trial registration:**

PROSPERO registration # CRD42015017182.

**Electronic supplementary material:**

The online version of this article (10.1186/s12889-017-4754-1) contains supplementary material, which is available to authorized users.

## Background

There are increasing disparities in socioeconomic status within and across populations [1]. These disparities are intricately linked to inequalities in health and health-related outcomes [2–4]. Education is an important indicator of socioeconomic status (SES), as it is completed early in the life course, usually fixed after a certain age, and associated with subsequent income, employment, social networks, and behaviors [5]. Increasing educational attainment is associated with better health outcomes and delayed mortality [5–8]. Therefore, an understanding of the specific role of education as both a potential lever to reduce socioeconomic inequality and to improve health outcomes is important for policy makers and public health officials. Despite the large body of literature supporting the association between education and health, the specific magnitude of this relationship is unknown.

Understanding the independent association between education and mortality is complex. While educational attainment is influential in determining occupation and income, separating the unique and specific educational effect from the broader socioeconomic relationship between education and health is challenging. Increasing education impacts availability of resources, self-efficacy, and opportunities for both individuals and communities [5]. Many of the suggested mechanisms through which education and SES impact health are often intricately linked to education itself. It is difficult to ascertain if education entirely operates through these mediation pathways, or if there is a separate benefit linked to education after accounting for these contributing factors. Education also has differential effects across the schooling spectrum. For example, Montez et al. have shown that the mortality association with education does not merely equate to sum of years in school, but is related to achieved credentials [9]. However, education provides more than a certificate. To understand the full impact of education on mortality, it is important to consider not only the material rewards that flow from the certification necessary for employment, but also the cognitive resources that may be more difficult to measure [10].

The complexities in establishing the independent and adjusted effects of education have made it difficult to synthesize and quantify the precise relationship between increasing educational attainment and mortality. Understanding education’s independent effects are critically important as policy makers design and fund resources to mitigate health disparities, particularly disparities in early mortality. To bridge this gap, we conducted a comprehensive systematic review of the education-mortality literature. In doing so, we discovered significant variation in study designs, populations, and outcomes. Because of this variation, many of the studies evaluating the association between education and mortality are not directly comparable. Therefore, we constructed a novel taxonomy of all studies evaluating education and mortality. Our taxonomy groups studies by similarity in study design and provides the necessary framework to formally compare studies so as to quantify the relationship between increasing education and mortality reduction.

## Methods

We followed PRISMA recommendations when performing this review (Additional file 1: Figure S1) [11]. The study protocol was registered with the PROSPERO register of systematic reviews (registration # CRD42015017182).

### Search strategy

We searched PubMed and Embase from database inception (1950, 1947 respectively) to April 2016 to identify relevant articles examining the association between educational attainment and mortality. Articles were retrieved from electronic searches, as well as by a manual hand search of bibliographies within eligible papers. We also contacted experts in education and mortality research for additional unpublished articles. Studies involving child mortality, maternal mortality, or perinatal mortality were excluded, as this review is focused on the association of completed education with mortality. We excluded conference proceedings and abstracts. The search was limited to articles published in the English language. The initial database search resulted in 1711 titles. Details regarding the search strategy are available in the Additional file 2: Figure S2.

### Study selection and eligibility criteria

**Fig. 1:**
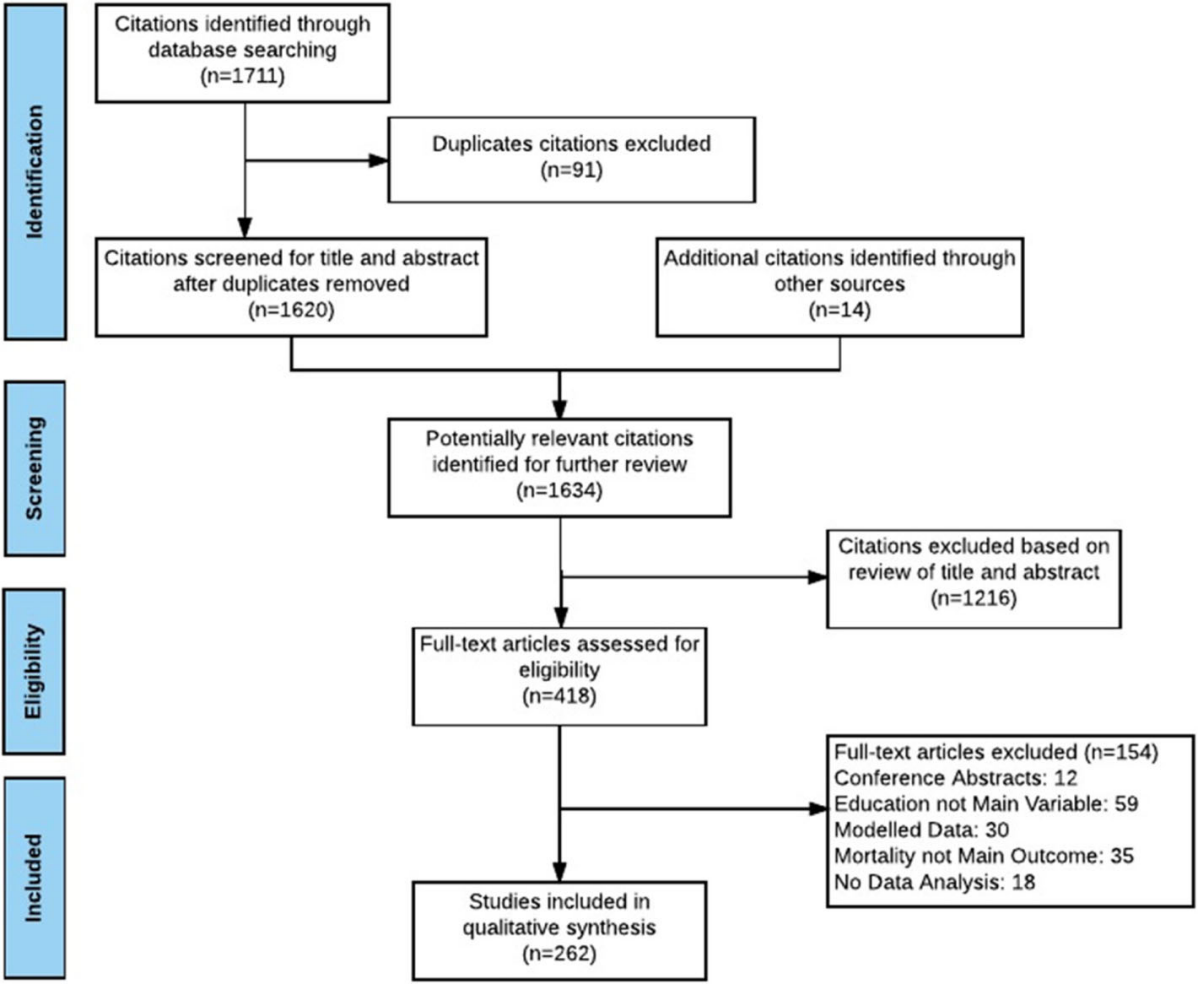
PRISMA Study Flow Diagram. Flow chart for included and excluded studies for this review

Three reviewers (RP, MH and EB) independently screened all titles and abstracts identified from the literature search for relevance. Full paper manuscripts were then independently reviewed for eligibility. Any difference of opinion was adjudicated by a fourth author (VC). Studies were included if they (a) reported mortality as a main outcome; (b) included educational attainment as an independent variable when performing statistical analyses; (c) represented a primary data analysis of individual level data, as opposed to aggregate data, such as county or national level data; and, (d) reported the independent association of educational attainment on mortality. We excluded studies if they: (a) were not original research (i.e., discussions, editorials, reviews, or descriptions of causal pathways); (b) were validation studies of explanatory models; (c) used estimated or simulated population data, such as life tables or life expectancy; or (d) included education as a confounder, not a main predictor in the analysis (Fig. 1). After applying these criteria, 262 studies remained and were included in the review.

#### Data extraction and quality assessment

Data from studies meeting inclusion criteria were entered by two authors (RP and MH) into an electronic study abstraction template modeled after the Cochrane Collaboration [12]. The abstraction template included how the educational attainment variable was used: either as a continuous, categorical or dichotomous variable. Quality assessment and risk of study bias were assessed using the Downs and Black tool as recommended by the Cochrane Collaboration [12]. This tool produces a score (max = 14) for methodological quality by asking 14 questions in four categories (reporting, external validity, internal validity/bias, and internal validity/confounding). As none of the included studies were randomized due to the cohort and cross-sectional nature of the evidence, questions regarding randomization were removed from the tool for all studies. Studies receiving a score of ten or above were considered to be at low risk of bias whereas studies scoring nine or below were considered to be at high risk of bias. Studies at high risk of bias were excluded from the presented results.

### Taxonomy Development & Validation

We used an inductive thematic and analytic approach to develop a taxonomy to describe, classify and assess the evidence evaluating the association between educational attainment and mortality [13]. All included studies were classified according to this taxonomy and placed into specific domains. As full text review was performed during abstraction, the authors used an iterative approach to determine domains for the final taxonomy. A priori, domains were developed based on study design (e.g., longitudinal survey data collection, census), analytical approach (e.g., multivariate regression, cox proportional hazards, age-standardized mortality rates), and outcome measures (e.g., all-cause mortality, cause-specific mortality, mortality rate changes) specific to each study. Each article was evaluated for these items and assigned a primary domain.

Final domains for classifying studies of the association between educational attainment and mortality are as follows: (1) all-cause mortality studies, (2) outcome-specific mortality studies, (3) studies that proposed an explanatory pathway, and (4) studies that reported trends in the education-mortality association over time. The all-cause mortality category was comprised of studies primarily focused on the association between education and all-cause mortality using nationally and internationally representative samples. These studies also included evaluations of the association of socioeconomic status and mortality, but only if education was included as an independent predictor and effects attributable to education were reported in study results. Outcome-specific mortality studies comprised those examining the association between education and cause-specific mortality (e.g., death from cardiovascular disease or cancer). Explanatory pathway studies explicitly evaluated the roles of behavioral, social or environmental factors that might mediate the education-mortality relationship (e.g., whether differences in smoking or alcohol consumption by educational attainment accounted for the association of education and mortality). We included studies assessing behavioral, psychosocial, cognitive, genetic and environmental mediators of the education-mortality relationship in this domain. Finally, studies included in the trends over time domain were those that examined population level changes in the association between mortality and educational attainment over time. Compared to other categories, these studies were unique in that their statistical analyses were presented as a change in hazard rate, risk rate or mortality rate between two specified periods.

When studies met criteria for two or more domains, we assigned a primary domain based on the (1) methodology and (2) reported primary outcome. For instance, a study evaluating changes in lung cancer mortality over time was primarily categorized as a trend over time study based on the statistical analysis, but secondarily categorized as an outcome specific-study as cause-specific mortality was measured. For the purposes of this systematic review, we included each study only once in its primary categorization. The overlap of assigned domains was evaluated for common themes in the study design and ultimately collapsed into final consensus categories [14].

#### Results of the education/mortality association

The nature of the association between mortality and education was defined as being either “significant”, “mixed,” or “not significant” for each included study. These determinations were made independently by authors (EB, MH) based on the presence of a statistically significant association (*p* ≤ 0.05) between education and mortality in the presented results and tables. Studies where all results and tables had a statistically significant association between mortality and education (ie *p* ≤ 0.05 or CI did not cross 1) were considered to have a “significant” outcome. Significant studies included those with either direct or inverse relationships between education and mortality [15]. Included studies were considered to have a “mixed” association if some of the final results included a statistically significant association (p ≤ 0.05) between education and mortality, while some of the results did not maintain statistical significance (*p* > 0.05). For example, many of the studies reported results stratified by gender. In doing so, the results for the female group may not have shown a statistically significant association between education and mortality (*p* > 0.05), while results for the male group remained significant [16–19]. Studies with no significant association were those with no statistically significant association between the education variable and the mortality in all results (*p* > 0.05).

#### Statistical analysis

Owing to substantial heterogeneity across the included studies, formal meta-analyses were not attempted. Rather, descriptive statistics were used to summarize the findings of the included studies and the proposed taxonomy.

This study was deemed exempt from the University of Michigan Institutional Review Board.

## Results

From the 1711 titles generated from our initial search, 418 papers were included for full text review and 262 met final inclusion criteria (Fig. 1). A total of 68 (26.0%) studies were included in the all-cause mortality domain, 89 (34.0%) in the outcome-specific mortality domain, 51 (19.5%) in the explanatory pathways domain, and 54 (20.6%) in the trends over time domain. Of all the studies included, 96 (36.6%) showed a statistically significant association between educational attainment and mortality (Table 1).

**Table 1:** Characteristics of included studies (By taxonomy)

Primary Domains^a^					
	All-Cause Mortality	Outcome Specific	Trends Over Time	Explanatory Pathways	Total
	n = 68(%)	n = 89(%)	n = 54 (%)	n = 51 (%)	262
Year of publication					
Before-1990	4(5.9)	2(2.2)	1(1.9)	1(2.0)	8(3.1)
1991–2000	10(14.7)	15(16.9)	2(3.7)	6(11.8)	33(12.6)
2001–2010	40(58.8)	31(34.8)	20(37.0)	20(39.2)	111(42.4)
2011-present	14(20.6)	41(46.1)	31(57.4)	24(47.1)	110(42.0)
Study Population					
Survey/Longitudinal	28(41.2)	39(43.8)	3(5.6)	26(51.0)	96(36.6)
Census data	39(57.4)	38(42.7)	51(94.4)	24(47.1)	152(58.0)
Other	3(4.4)	14(15.7)	0(0.0)	1(2.0)	18(6.9)
Study Design					
Cohort	48(70.6)	73(82.0)	25(46.3)	42(82.4)	188(71.8)
Cross-sectional	19(27.9)	15(16.9)	28(51.9)	6(11.8)	68(26.0)
Mixed methods	1(1.5)	1(1.1)	1(1.9)	3(5.9)	6(2.3)
Region of Study					
North America	27(39.7)	24(27.0)	19(35.2)	9(17.6)	79(30.2)
U.S.	24(35.6)	21(23.6)	19(35.2)	9(17.6)	73(27.9)
South America	1(1.5)	2(2.2)	2(3.7)	1(2.0)	6(2.3)
Europe	30(44.1)	51(57.3)	29(53.7)	35(68.6)	145(55.3)
Asia	11(16.2)	14(15.7)	4(7.4)	6(11.8)	35(13.4)
NZ/Australia	0(0.0)	3(3.4)	2(3.7)	1(2.0)	6(2.3)
Africa	0(0.0)	0(0.0)	0(0.0)	0(0.0)	0(0.0)
Number of Countries					
Single	65(95.6)	78(87.6)	50(92.6)	47(92.2)	240(91.6)
Multiple	3(4.4)	11(12.4)	4(7.4)	4(7.8)	22(8.4)
Educational Association					
Significant	24(35.3)	35(39.3)	26(48.1)	11(21.6)	96(36.6)
Mixed	30(44.1)	27(30.3)	16(29.6)	27(52.9)	100(38.2)
Not Significant	15(22.1)	27(30.3)	11(20.4)	13(25.5)	66(25.2)
Stratified by age	30(44.1)	23(25.8)	19(35.2)	10(19.6)	82(31.3)
Stratified by race	8(11.8)	7(7.9)	11(20.4)	4(7.8)	30(11.5)
Stratified by gender	40(58.8)	39(43.8)	50(92.6)	32(62.7)	161(61.5)
Conditioned on SES	11(16.2)	5(5.6)	2(3.7)	3(5.9)	21(8.0)

^a^Totals for some categories may exceed 100% as articles are able to fall into more than one category

To validate our taxonomy, (and ensure no substantial overlap between categorization of articles), we created a validation matrix (Table 2). The validation matrix quantified the number of articles with primary and secondary categorizations and also evaluated the studies categorized in more than one domain by assessing the off-diagonal. Our categorization resulted in 214 (81.7%) of all articles categorized under a single domain, with 48 (18.3%) having a secondary domain. This suggests our identified domains are not redundant and delineate distinct study types.

**Table 2:** Validation matrix for taxonomy development

Second Category					
	All-Cause Mortality	Outcome Specific	Trends Over Time	Explanatory Pathways	Total
Main category					
All-Cause Mortality	61	0	2	5	68
Outcome Specific	0	75	9	5	89
Trends Over Time	4	14	35	1	54
Explanatory Pathways	4	3	1	43	51
Total					262

### Results by taxonomy category

#### All-cause mortality

Of the 262 studies included in our systematic review, 68 (26.0%) examined the association between education and all-cause mortality. This domain included the largest number of U.S. based studies (*n* = 24, 35.6%); other regions included Europe (*n* = 30, 44.1%), Asia (*n* = 11, 16.2%) and South America (*n* = 1, 1.5%). Of the 68 studies included in this domain, 24 studies (35.3%) demonstrated a statistically significant association between educational attainment and mortality. The majority of studies in this domain showed statistically mixed results (*n* = 30 studies, 44.1%), analysis stratified by gender, race/ethnicity, or age often contributed to non-significant associations between educational attainment and mortality for some studies, especially those that used education as a categorical variable. Subgroup analysis varied widely in this cohort of studies, including subgroup analysis based on geographic location, cause of death, biometric data, or socio-demographics. Loss of statistical significance between educational attainment and mortality was also common in the studies that performed study-specific subgroup analysis. A statistically significant association was reported when comparing groups with the highest versus the lowest educational attainment while those with intermediate levels of educational attainment frequently had no statistically significant mortality benefit when compared to those at the extremes. This was also observed in studies that stratified by age, where age effects in older subgroups trended towards a non-significant association between educational attainment and mortality and in studies that included cause-of-death subgroup analysis. Additional file 3: Table S1 provides details on all of the studies categorized as all-cause mortality.

#### Outcome-specific mortality

This was the largest domain in the taxonomy and included 89 (34.0%) studies whose dependent variable was cause-specific mortality. Examples of outcome-specific studies include those evaluating the association between educational attainment and death from cancer, cardiovascular disease, accidental deaths, alcohol and drug-related deaths, stroke, post-operative mortality, diabetes-related diseases, dementia, rheumatologic disease, liver and gastrointestinal disease, end stage kidney disease, infectious diseases, or trauma (Additional file 3: Table S2). The cause of death in 25 of the studies in this domain was cancer (28.1%). Many of the papers included covariates beyond age, gender, education and other demographic or socioeconomic indicators. For instance, studies evaluating cardiovascular disease also often adjusted for confounders such as body mass index, systolic blood pressure, glycosylated hemoglobin or lipodensity protein level. The majority of studies in this domain showed a statistically significant association between education and mortality (*n* = 35/89 39.3%), while 27 studies (30.3%) had mixed results. Common reasons for loss of statistical significance in outcome specific studies included subgroup analysis by different cancer types, use of education as a categorical variable or analysis stratified by gender.

#### Trends over time

Fifty-four studies (20.6%) evaluated differences or rate changes between education and mortality over time. While these studies often used similar statistical methods, (e.g., changes in age-standardized mortality rates) to assess the education-mortality gradient, the final results in this domain are unique in that they report the change in association between time periods rather than a ratio or risk.

The majority of trend over time studies were published after 2010 (*n* = 31, 57.4%). Additionally, these studies also included the largest stratified analysis by gender (*n* = 50, 92.6%). The trends over time domain had the largest number of papers with a secondary domain assignment (*n* = 19, 35.2%). Of those with a secondary domain, the largest subgroup included papers with outcome-specific mortality (*n* = 14, 25.9%). Compared to any of the other domains, papers in the trend domain most often reported a statistically significant association between educational attainment and mortality (*n* = 26/54, 48.1%). Sixteen of the studies demonstrated statistically mixed results (*n* = 16/54, 29.6%). The reasons for loss of statistical significance in some results included subgroup analysis by cancer type, by age group, and by gender (Additional file 3: Table S3).

#### Explanatory pathways

This domain included 51 studies (19.5%) that evaluated the causal pathway linking higher educational attainment to lowered mortality risk. Included studies assessed the effect of family educational attainment, spousal educational attainment, smoking, alcohol use, IQ, neighborhood and geographic exposures on overall mortality (Additional file 2: Table S4). Notably, this domain included the fewest studies to show a statistically significant relationship between educational attainment and mortality (*n* = 13, 25.5%). The largest proportion of studies had mixed results (*n* = 27, 52.9%). Commonly, stratification by gender, age, study region, and cause of death resulted in a loss of statistical significance in the final presented data. In some studies, controlling for behavioral mediators, such as tobacco use also resulted in a loss of statistical significance.

### Risk of bias

Risk of bias for all studies was evaluated using a modified Downs and Black Scale, as recommended by the Cochrane Collaboration for non-randomized studies [20]. The mean score of all included studies was 12.0 (range 0–14, SD ±1.42), corresponding to low risk of bias. Studies in the population level association domain had a mean score of 11.9 (range 9–14, SD ±1.37). Outcome specific studies had a mean score of 12.0 (range 8–14, SD ±1.35). Trend studies had a mean score of 11.5 (range 8–14, SD ±1.61). Explanatory pathways studies had a mean score of 12.2 (range 10–14, SD ±1.22). Thirteen studies deemed to be at high risk of bias, with a modified Downs and Black score of nine or less; over half of these (*n* = 7) were in the trends over time domain.

## Discussion

While some studies suggest a significant association between educational attainment and mortality [5–8, 21, 22], others suggest the association is not as robust [23–25]. To shed new light on this topic, we conducted the most comprehensive review of the literature to date. We found marked variability in study design, populations studied and results regarding the education-mortality association. Of the 262 studies included in our review, 66 (25.2%) demonstrated no statistically significant association between education and mortality in their final results. One hundred (38.2%) showed mixed results, where certain subgroups did not show a statistically significant association between education and mortality. These findings suggest that difficulties in ascertaining a more precise association between educational attainment and health outcomes may exist because of the substantial heterogeneity in study design, outcomes and methods across studies.

This study aimed to create a framework upon which future evaluations can be done to ascertain and quantify the relationship between increasing educational attainment and mortality. Given the large volume of literature, few reviews and meta-analyses exist [8, 22, 26, 27]. The education-mortality relationship is difficult to definitively capture due to the endogenous nature of the mechanistic pathways through which education serves to improve health and reduce mortality. However, understanding the relative importance of the various mechanisms through which increasing education prevents early mortality, and the independent effect of education in delaying death, has significant consequences for both health and social policy with respect to determining the best targets to improve overall health and reduce early mortality.

Our results do not definitively suggest that education alone can explain mortality differentials. While education is an individual resource, it is embedded across the lifespan and exists within a framework of larger societal characteristics [7]. Education itself differs in quality and timing across populations, which likely influences its varying relationship to health outcomes [28]. As a fundamental cause of broader health disparities, education serves an important role in reducing inequality across generations [29]. Yet it is intricately linked to factors along the pathway to mortality. However, the importance of distinguishing education’s independent protective effects from the larger pathways through which education results in delayed death has significant policy implications. When public health officials and policy makers consider interventions to reduce mortality inequalities at the population level, it is important to understand where dedication of resources might have the biggest impact, and over what time course. If education has a large independent effect on delaying mortality after adjusting for mediators, targeting resources to improving and increasing accessibility to traditional education programs would translate into reduced mortality disparities with the potential for long-lasting impact, albeit over a much longer time course. However, if evaluating the education effect suggests that educational benefits are largely explained via the mediating pathways through which education improves health, with minimal independent effects of education, then implementing programs aimed at specific populations with low education to improve health literacy, behaviors, access, or other targets could most efficiently mitigate health and mortality disparities.

It is difficult to control for reverse causality inherent in in studies that evaluate the education-mortality association. That is, the association between education and mortality may reflect decreased educational attainment due to pre-existing or childhood illness that would increase risk of death and decrease the likelihood of advanced educational attainment [30]. Additionally, the inclusion of largely observational studies (cohort and cross-sectional design) limits insights into causal relationships. The literature evaluating the education-mortality association acknowledges this limitation. Analysts have used changes in schooling laws as a natural experiment to ascertain and support the causal association of education on mortality [21, 23]. A prior meta-analysis of 22 studies of European natural experiments in schooling reforms have suggested that education has a small but independent effect that varies across age groups and gender [22]. Finally, our determination of the overall education-mortality association was based upon statistical significance. While this is a commonly accepted metric to propose a meaningful association and was able to be successfully applied to the majority of studies included in this review, it is unclear whether statistically significant results in these studies have important clinical relevance.

Despite these limitations, our review also has several strengths. First, to our knowledge, this is the largest systematic review to evaluate the education-mortality association in the evidence-based literature to date. An earlier systematic review conducted on the education-mortality association limited the dates of inclusion and implemented narrower inclusion criteria, yielding a more homogenous and limited sample [8]. Second, we utilize methodological, analytic and population level heterogeneity to develop a taxonomy that can be used to further understand the education-mortality association. Our review included 262 articles evaluating mortality by educational attainment, yet due to study heterogeneity, we were unable to quantify the association between these two factors. This taxonomy will allow for future meta-analyses to be performed across similarly designed studies, and can provide a quantitate association of the education/morality relationship within identified domains. Third, we organize and present our results into the domains of all-cause mortality, outcome-specific mortality, explanatory pathways, and trends over time. By looking within each domain, we propose a framework within which further work can be done to better quantify the relationship between education and mortality across populations, and to identify opportunities and gaps in the current evidence. While many individual studies within each domain demonstrate a positive association between increased educational attainment and reduced mortality, future work to pool data across studies of different populations may demonstrate that no global relationship exists.

## Conclusions

Understanding and quantifying the benefits of education has important public health implications. As continuing globalization and economic development changes the nature and importance of formal education, it is important to understand how much investment in increasing educational attainment can reduce growing mortality disparities. Education is often seen as an important social lever to ameliorate inequalities. We propose a novel taxonomy that can leverage existing data to better understand how inequalities in educational attainment translate into inequality in early mortality. Building on this framework, a similar methodology could be employed to the education and health literature more broadly, as the themes identified in this review are not unique to mortality inequality. Further evaluation into the explanatory pathways domain specifically - including meta-analyses of behavioral or social network level factors - can have significant policy relevance by highlighting factors that may be contributing to broader health inequalities, but amenable to targeted interventions.

As public resources are limited, perhaps we can utilize the independent benefits of education as a source of motivation to further emphasize the public health importance of good schooling as a matter of life or early death.

## Additional files

Additional file 1: Figure S1. Search Strategy. Screen shot of pubmed search strategy for conducting the review. (DOCX 140 kb)
**Additional file 2: Figure S2.**

Additional file 3: Tables S1-S4. These are the 4 appendix tables of all included studies in the review, alphabetically organized, and grouped by domain. **Table S1.** All Cause Mortality; **Table S2.** Outcome Specific; **Table S3.** Trends Over Time; **Table S4.** Explanatory Pathway. (DOCX 127 kb)
