# Supplementary figures and images for: Increasing educational attainment and mortality reduction: a systematic review and taxonomy
Source: BMC Public Health. 2017 Sep 18;17:719. doi: 10.1186/s12889-017-4754-1 (PMC5604174; doi:10.1186/s12889-017-4754-1)

**Figure S1**: Search Strategy


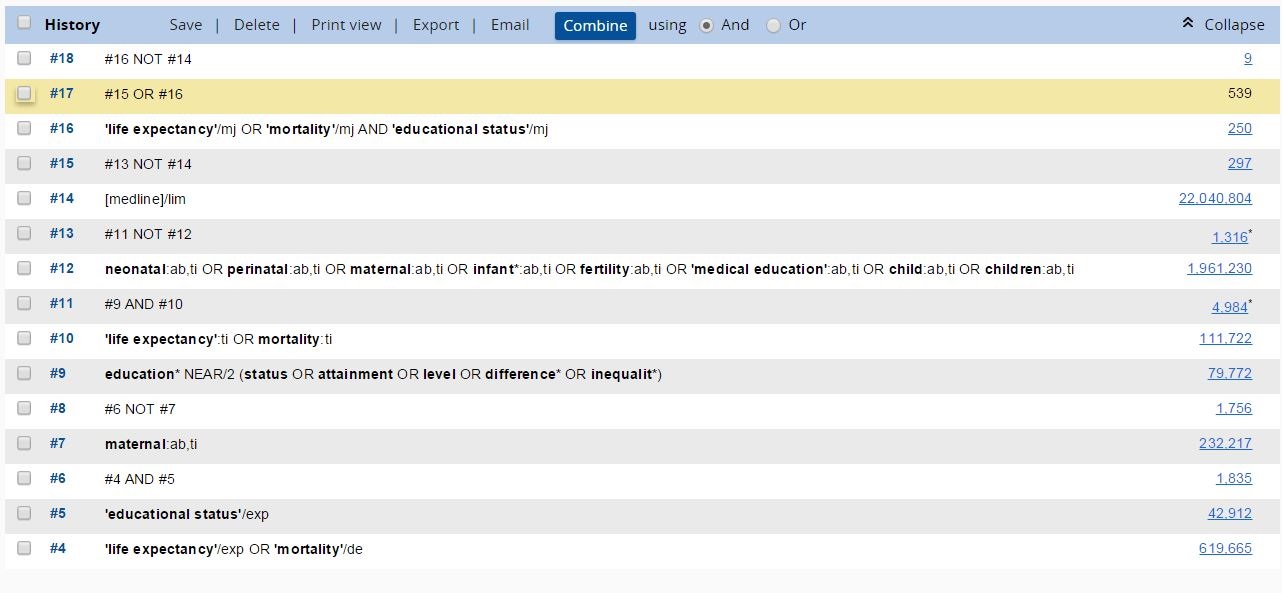

Supplement: Supplementary file 1 — Search Strategy. Screen shot of pubmed search strategy for conducting the review. (DOCX 140 kb) Additional file 2: Figure S2. [file 12889_2017_4754_MOESM1_ESM.docx]
