# Supplementary material for: Increasing educational attainment and mortality reduction: a systematic review and taxonomy
Source: BMC Public Health. 2017 Sep 18;17:719. doi: 10.1186/s12889-017-4754-1 (PMC5604174; doi:10.1186/s12889-017-4754-1)
Supplement: Supplementary file 2 — These are the 4 appendix tables of all included studies in the review, alphabetically organized, and grouped by domain. Table S1. All Cause Mortality; Table S2. Outcome Specific; Table S3. Trends Over Time; Table S4. Explanatory Pathway. (DOCX 127 kb) [file 12889_2017_4754_MOESM3_ESM.docx]

**Table S1:** Description of articles included in All-Cause Mortality Domain, alphabetically. (N=68)

| Author (yr) | Study Region | Aim | Study Population | | Study Design | Education Variable | Conditioned on SES | Quality Ranking Score | | Second Category | Education Effect | Stratified by Race | Stratified by Age | | Stratified by Gender | Predictor Variables |
| --- | --- | --- | --- | --- | --- | --- | --- | --- | --- | --- | --- | --- | --- | --- | --- | --- |
| Albouy, V. et al (2009) | France | We wish to add the French case to the existing literature, and test the existence of a causal mechanism between education and mortality on French data. | | census | cohort | continuous | no | 11 | None | | not significant | no | no | no | | school reform, school leaving age |
| Backlund, E. et al (1999) | United States | To compare and contrast the functional forms of the relationships of education and income with mortality. | | census | cohort | categorical | no | 13 | None | | significant | no | no | yes | | income, education, age, sex, race, household size, marital status, employment status |
| Bessudnov, A. et al (2012) | Russia | We investigate the role of class and perceived status as bases of social inequalities in Russian mortality. | | survey | cohort | categorical | yes | 12 | None | | not significant | no | no | n/a | | household income per capita, marital status, educational attainment, ethnicity, status |
| Bopp, M. et al (2003) | Switzerland | The aim of this paper is to show for the first time mortality differentials by level of education for Swiss men and women. | | census | cohort | categorical | no | 14 | None | | significant | no | yes | yes | | age, sex, education |
| Borrell, C. et al (1999) | Spain | To assess mortality differences according to educational level using individual-based information in the cities of Madrid and Barcelona. | | census | cross sectional | categorical | no | 12 | None | | mixed | no | yes | yes | | age, sex, city of residence, educational level and cause of death |
| Cacciani, L. et al (2015) | Italy | We analyzed data from a large cohort of residents in Rome followed-up between 2001 and 2012 to assess the relationship between individual education and mortality. We distinguished five causes of death and investigated the role of age, gender, and birthplace. | | census | cohort | categorical | no | 12 | None | | significant | no | yes | yes | | age, gender, and birthplace, and educational level attained, marital status, unemployment |
| Choi, A.I. et al (2011) | United States | We conducted this study to determine the association between educational attainment with chronic diseases and survival in a national sample of participants of a health screening program. | | survey | cohort | categorical | no | 11 | None | | significant | no | yes | yes | | self-reported educational attainment, socio-demographic characteristics, and medical information: |
| Comstock, G.W. et al (1978) | United States | To calculate death rates from major causes for a sizable number of individuals according to their educational status after removing the effects of several other important variables by multiple adjustments. | | census | cohort | categorical | no | 11 | None | | mixed | no | no | no | | race, sex, age, marital status, education, housing facilities, smoking, and church attendance |
| Doornbos, G. et al (1990) | Netherlands | In the present study, the relationship between educational level and differential mortality is investigated and confounding of this relationship by other related characteristics will be taken into account. | | survey | cohort | categorical | no | 12 | None | | significant | no | no | n/a | | education, dichotomized health, BMI |
| Dupre, M.E. (2007) | United States | This research addresses this in consistency by differentiating individual-level changes in health from those occurring at the aggregate level due to selective mortality. | | survey | cohort | categorical & dichotomized | no | 11 | None | | mixed | no | no | no | | age, age of onset, education level, sex, race, gender, comorbidities |
| Elo, I.T. et al (1996) | United States | This paper examines educational differentials in adult mortality in the United States, and uses a recently-released data set, the National Longitudinal Mortality Survey (NLMS). | | census | cohort | categorical | no | 11 | None | | mixed | no | yes | yes | | sex, age and year of school completed, race, region of birth, income, marital status, metropolitan residence and number of household members |
| Erikson, R. et al (2009) | Sweden | Mortality is strongly associated with education. We present relative death risks of men and women in 12 educational/occupational groups in Sweden today, with a focus on individuals with higher education. | | census | cross sectional | categorical | yes | 12 | None | | mixed | no | no | yes | | sex, age, profession (12), education |
| Everett, B.G. et al (2013) | United States | The analyses presented in this paper improve upon existing research on the relationship between education and mortality using updated mortality information; disaggregating our analysis by cohort, race/ethnicity, and gender; and employing a more flexible modeling approach. | | census | cross sectional | categorical & continuous | no | 11 | Trends | | mixed | yes | no | yes | | age, gender, race/ethnicity |
| Faeh, D. et al (2010) | Switzerland | Our aim was to analyze educational inequalities in all-cause and cause-specific mortality in the two Swiss regions and to compare this with inequalities in behavioral risk factors and self-rated health. | | census | cohort | categorical | no | 13 | None | | significant | yes | yes | yes | | SES, risk factors (alcohol, physical activity, BMI, self-rated health and smoking) |
| Fujino, Y. et al (2005) | Japan | To determine the impact of socioeconomic status on health among the Japanese population. | | survey | cohort | categorical | no | 13 | None | | mixed | no | no | yes | | education, age, smoking status, alcohol consumption, job status, and type of job |
| Hardarson, T. et al (2001) | Iceland | Our objective was to estimate the relationship between educational level and coronary artery disease (CAD), mortality and all-cause mortality. | | survey | cohort | categorical | no | 13 | None | | significant | no | no | yes | | age at first examination, year of first examination, height, weight, total cholesterol, triglycerides, 90 min blood sugar and current smoking |
| Hirokawa, K. et al (2006) | Japan | The objective of this study was to examine educational levels and employment status as independent determinants of overall and cause-specific mortality in a Japanese population. | | survey | Cohort | categorical | no | 11 | None | | not significant | no | yes | yes | | age, gender, marital status, alcohol consumption, smoking status, education, occupation, dietary habits, BMI, menopausal |
| Huisman, M. et al (2005) | Finland, Norway, England, Wales, Belgium, Switzerland, Austria, Italy, Spain | Our aim was to broaden the scope of the evidence base for European public-health policies, by assessing the contribution of specific causes of death to differences in mortality by socioeconomic level. | | census | cohort | dichotomized | no | 12 | None | | significant | no | yes | yes | | age, educational levels, sex, country |
| Hurt, L.S. et al (2004) | Bangladesh | To examine socioeconomic gradients in mortality in adult women and their husbands in Bangladesh, paying particular attention to the independent effects of the educational status of each spouse. | | census | cohort | categorical | no | 13 | None | | significant | no | no | yes | | sex, education, religion, age, social status, occupation, are of residence, marital status, reproductive history |
| hypertension detection and follow-up program cooperative group (1987) | United States | To determine if the adverse effect of low educational level was uniformly present in HDFP, or if the stepped care (SC) program diminish this effect among those randomized to SC? What effect did degree of blood pressure control have on mortality, and was this independent of educational level? | | survey | cohort | categorical | no | 10 | None | | mixed | yes | no | no | | age-sex-race, baseline medical status, and blood pressure control during the trial, and education |
| Ito, S. et al (2008) | Japan | The objective of this study was to examine the association between educational level and health status in a population based, prospective cohort study in Japan. | | survey | cohort | categorical | no | 13 | None | | mixed | no | no | yes | | sex, age, public health center area, behavioral factors, preventative care, and healthy eating |
| Jemal, A. et al (2008) | United States | To analyze national data to measure the number of deaths associated with lower education among working-aged adults by race or ethnicity. Furthermore, to examined the relationship of education to cause-specific and all-cause mortality in the three largest racial or ethnic groups in the United States using national data. | | census | cross sectional | categorical | no | 12 | None | | significant | yes | no | yes | | age, gender, and race/ethnicity |
| Kalist, D.E. et al (2007) | United States | The authors used duration analysis to examine the longevity of Major League Baseball players. | | other | cohort | dichotomized | no | 9 | None | | significant | no | no | n/a | | dominant hand, BMI, birth city, race, hall of fame ballot, MLB career, education level, birth year |
| Kaplan, R. M., et al. (2015) | United States | In this article, we report analyses of the relationship between educational attainment and life expectancy using a large heterogenous cohort created for the national Reasons for Geographic and Racial Differences in Stroke (REGARDS) study. | | survey | cohort | categorical | no | 13 | None | | mixed | yes | yes | no | | demographic factors (age, race, and sex), income, cardiovascular risk factors (hypertension, diabetes, smoking, dyslipidemia, and body mass index [BMI]), and behavioral factors (alcohol use, exercise, and perceived stress) |
| Khang, Y.H. (2004) | South Korea | To examine age- and cause-specific socioeconomic mortality differentials in a sample that includes both men and women representative of the Korean population. | | census | cohort | categorical | no | 14 | None | | not significant | no | yes | yes | | age, sex, education |
| Khang, Y.H. (2005) | South Korea | To examine relationship of education, occupation, and income with mortality risk, the 1998 National Health and Nutrition Survey data were linked to data on mortality. | | survey | cross sectional | categorical | no | 12 | None | | not significant | no | yes | yes | | age, sex, education |
| Kiuila, O. et al(2007) | United States | Our basic objective is to confirm and to explain a key result of the literature on the social determinants of health: The socioeconomic mortality gradient is much steeper at younger ages than it is above the age of 65. | | census | cohort | categorical | no | 10 | None | | mixed | no | yes | no | | demographic factors, social and economic factors, behavioral factors, and health factors |
| Koch, E. et al (2010) | Chile | To test the hypothesis that an inverse association exists between socioeconomic position and all-cause mortality in a developing country in Latin America. | | survey | cohort | categorical | yes | 12 | None | | significant | no | no | no | | Education level, income quarters, behavioral and biological risk factors for chronic diseases |
| Kravdal, O. (2009) | Norway | The intention was to find out whether there was an association between the socioeconomic resources in a small neighborhood ("basic statistical unit"; BSU) and individual mortality, net of individual resources, and whether this association differed between municipalities including a quite large city and others. | | census | cohort | categorical | no | 10 | None | | mixed | no | yes | no | | age, education, sex, characteristics of BSU & municipality |
| Kulhanova, I. et al (2014) | Netherlands | Using new facilities for linking large databases, we aimed to evaluate for the first time the magnitude of relative and absolute educational inequalities in mortality by sex and cause of death in the Netherlands. | | survey | cohort | categorical | no | 11 | None | | significant | no | no | yes | | sex, cause of death, age, education |
| Kunst, A.E. et al (1994) | Netherlands, Denmark, Norway, Sweden, Finland, England and Wales, France, Italy, United States | This study addresses the question of whether inequalities in premature mortality related to educational level differ among countries. | | survey & census | cohort | categorical | no | 10 | None | | mixed | no | yes | no | | age, country, mean age at death |
| Lager, A.C. et al (2012) | Sweden | Children in a number of municipalities were exposed to the reform and others were kept as controls, allowing us to test the hypothesis that education is causally related to mortality. | | other | cohort | categorical | no | 11 | None | | mixed | no | no | yes | | Education, sex, birth cohort, age |
| Liu, X. et al (1998) | Taiwan | The present research examines the impact of education on the mortality of older Taiwanese during a 4-year interval from April 1989 to April 1993. | | survey | cohort | categorical | no | 10 | Pathway | | not significant | no | no | no | | basic demographic characteristics (e.g., education, gender, age), occupational history, social relationships, health status, and health care utilization |
| Long, J.A. et al (2002) | United States | The objective of this study was to determine the relationship between social class markers—education, income, husband’s work history, and personal work history—and mortality in a cohort of older women, after adjusting for clinical and behavioral factors. | | survey | cohort | categorical | yes | 11 | None | | not significant | no | no | n/a | | education, income, husband's and personal work history, demographic data, clinical data, and behavioral data |
| Luo, Y. et al (2015) | China | This study examines the relationship between education and mortality, its underlying mechanisms, and its gender and age variations among older adults in China, using data from the 2002 to 2011 waves of the Chinese Longitudinal Healthy Longevity Survey. | | survey | cohort | categorical | no | 12 | Pathway | | not significant | no | yes | yes | | gender, education, ethnicity, residence, age, occupation, social relationships and activities, health behaviors, Health status |
| Lynch, S.M. (2003) | United States | To examine how cohort structures the influence of education on life-course health trajectories. | | survey & census | cohort | continuous | no | 12 | None | | mixed | no | no | no | | age, race, region, sex, self-rated health |
| Martinez, C. et al (2009) | Spain | The objective of this work is to estimate the relation between educational level and mortality from the leading causes of death in adult women in different age groups in a region of southern Europe, and to evaluate the contribution of these causes of death to inequalities in mortality. | | census | cross sectional | categorical | no | 11 | None | | mixed | no | yes | n/a | | education, age |
| Masters, R.K. (2012) | United States | To analyze how age, period, and cohort affect U.S. adult mortality risk between 1986 and 2006 vary by educational attainment. | | census | cross sectional | categorical | no | 12 | None | | mixed | yes | yes | yes | | race, age, sex, education |
| Menvielle, G. et al (2010) | France | The aim of this paper is to investigate educational inequalities in mortality by gender, age and causes of death in France, with a special emphasis on people aged 75 years and more. | | census | cohort | categorical | no | 12 | None | | mixed | no | yes | yes | | age, education |
| Montez, J.K. et al (2009) | United States | We examine gender differences in the gradients within the context of marriage to determine whether overall differences reflect gender differences in health behaviors or a greater influence of men’s education on spousal health. | | census | cross sectional | categorical | no | 10 | None | | significant | no | no | no | | marital status, race, educational attainment, sex |
| Mustard, C.A. et al (1997) | Canada | The objective of this study was to describe age-specific socioeconomic differentials in mortality and morbidity for a representative sample of a single Canadian province. | | census | cross sectional | categorical | yes | 12 | None | | mixed | no | yes | no | | the level of educational attainment, total household income, age, sex |
| Naess, O. et al (2005) | Norway | In this study we examine whether the contextual effect of educational level aggregated to the neighborhood on mortality risk could be explained by earlier life deprivation. | | census | cohort | categorical | yes | 10 | None | | significant | no | yes | no | | education, age, neighborhoods, rooms per household capita, type of dwelling, ownership, toilet, bath, and telephone in dwelling, early life deprivation |
| Osler, M. et al (2003) | Denmark | To examine the educational level in the area of living as a determinant of all-cause mortality, controlling for individual and other correlated contextual factors. | | survey | cohort | categorical | no | 13 | None | | mixed | no | no | no | | educational attainment, local area unemployment, income share, and household composition, behavioral factors, the proportion of households with children, rates of unemployment, and the median share of income |
| Pensola, T.H. et al (2002) | Finland | The aim of the study is to examine the effects of parental class, own education and social class on mortality by cause of death among young men. | | census | cohort | categorical | yes | 13 | None | | significant | no | no | n/a | | educational attainment, social class, occupation, parental class, gender |
| Preston, S.H. et al (1995) | United States | We reconsider trends in educational mortality differentials using an alternative data source that is better suited to examining this issue, the NLMS. | | survey | cohort | categorical | no | 10 | None | | not significant | no | yes | yes | | gender, age, educational level, data source |
| Regidor, E. et al (2003) | Spain | The specific objective of this study was to evaluate the magnitude of the association between educational level and mortality from the leading causes of death and other specific causes of death in men and women, after adjusting for other socioeconomic variables. | | census | cross sectional | categorical | no | 13 | None | | mixed | no | no | yes | | education, gender, age, employment status, marital status, number of household members, place of birth, area-based deprivation category |
| Regidor, E. et al (2003) | Spain | We selected the population residing in Madrid (Spain), which has one of the highest rates of heroin use in Europe, in order to examine the association between education and mortality from various causes of death in young adults. | | census | cross sectional | categorical | no | 14 | None | | mixed | no | yes | yes | | education, gender, employment status, marital status |
| Regidor, E. et al (2005) | Spain | This study examines how education and employment situation contribute to the association between a classification of occupational class based on skill assets and mortality from different causes of death. | | census | cross sectional | categorical | yes | 14 | None | | significant | no | no | no | | education, occupation, employment status, |
| Regidor, E. et al (2015) | Spain | The objective of this study was to estimate the association between area-level socioeconomic context and mortality in Spain, using two different geographic aggregations. | | census | cross sectional | categorical | no | 13 | None | | not significant | no | yes | no | | age, sex, marital status, province and country of birth, place and residence time, education, employment status, professional status, occupation, economic activity of the company, ownership of housing, type of facilities available and number of vehicles |
| Regidor, E. et al. (2016) | Spain | To estimate educational inequalities in mortality in Spain and in three Spanish areas: Madrid, Barcelona, and the Basque country. | | census | cohort | categorical & dichotomized | no | 14 | None | | mixed | no | no | yes | | education, sex, city |
| Rehkopf, D.H. et al (2006) | United States | In this study, we used the newly available 2000 census population counts for education level cross-tabulated by age to report and compare, for the first time, the socioeconomic inequalities in mortality detected with individual-level education data and census tract area-based socioeconomic measures. | | census | cross sectional | categorical | no | 12 | None | | significant | no | yes | no | | education level, income level, age |
| Reques, L. et al (2014) | Spain | The objective of this study was to estimate inequalities in general and cause-specific mortality in the entire Spanish population and to calculate the contribution of the leading causes of death to the differences in total mortality. | | census | cohort | categorical | no | 13 | None | | mixed | no | yes | yes | | education, age, gender |
| Rogers, R.G. et al (2010) | United States | First, we present the first published estimates of U. S. adult mortality risk by detailed educational degree, including advanced postsecondary degrees. Second, to determine whether the association between educational degree and mortality risk differs across demographic subgroups, we examine these relationships both for the overall population and for specific cohort and gender subpopulations. | | census | cross sectional | categorical | no | 13 | None | | mixed | no | no | yes | | age, cohort, gender, race, marital status |
| Sabanayagam, C. et al (2012) | United States | In this context, we examined the independent effect of education and income on mortality by simultaneously adjusting for each other, in addition to the demographic, lifestyle and clinical factors. | | survey | cohort | categorical | yes | 13 | Pathway | | not significant | yes | no | yes | | age, education, gender, poverty-income ratio, race/ethnicity, marital status, smoking, ETOH, physical activity, BP, BMI, HDL |
| Saurel-Cubizolles, M.J. et al (2009) | France | The aim of this study was to compare inequalities in mortality (all causes and by cause) by occupational group and educational level between men and women living in France in the 1990s. | | census | cohort | categorical | no | 14 | None | | significant | no | no | yes | | educational level and occupational group, sex |
| Schwarz, F. (2007) | Austria | This paper not only aims to reveal mortality disparities, but also to provide possible explanations for disparities in major causes of death. | | census | cross sectional | categorical | no | 13 | None | | not significant | no | no | yes | | education, age, gender |
| Snowdon, D.A. et al (1989) | United States | Mortality among 306 Roman Catholic sisters (nuns) from Mankato, Minnesota, was assessed during the period 1936-1988. | | other | cohort | categorical | yes | 9 | None | | mixed | no | yes | n/a | | education, age, father's occupational class, mental status |
| Son, M. et al (2002) | Korea | We investigated mortality in the Korean working population aged 20–64 using registered death data from 1993 to 1997 obtained from the Korean National Statistics Office (NSO). | | census | cross sectional | categorical | yes | 10 | None | | significant | no | no | yes | | education, occupational class, gender |
| Steenland, K. et al (2002) | United States | We document in this paper which causes of death show the strongest relation with education and how these patterns have changed over time. | | survey | cohort | categorical | no | 14 | Trends | | significant | no | yes | yes | | education, age, smoking, BMI, menopausal status, diet, ETOH, HTN |
| Stirbu, I. et al (2010) | Europe | The objective of the present study is to estimate the magnitude of educational inequalities in avoidable mortality in different European countries and to prepare such an overview for a wide array of avoidable causes of death. The aim is to obtain indications on the role of the healthcare system in reducing socioeconomic inequalities in health. | | census | cross sectional & cohort | categorical & dichotomized | no | 11 | None | | significant | no | no | no | | education level, country |
| Sundquist, J. et al (1997) | Sweden | The aim of this study is to analyze the influence of different indicators of socio-economic position such as employment status, educational level of attainment, and housing tenure on mortality by studying a simple random sample of the Swedish population. | | survey | cohort | categorical | no | 13 | None | | significant | no | yes | yes | | ethnicity, marital status, working capacity, housing tenure, employment status, and educational level of attainment, age and sex |
| Tjepkema, M. et al (2012) | Canada | The objective of this study, therefore, is to examine cause-specific mortality rates by level of education to determine if the association between education and mortality differs by cause of death. | | survey | cohort | categorical | no | 11 | None | | mixed | no | no | yes | | education, age, sex |
| Tobiasz-Adamczyk, B. et al (2007) | Poland | The aim of this observational study was to assess the role of education and occupational status, including characteristics of occupational activity (e.g. health consequences of workplace exposure), and early retirement/disability pension [ER/DP]) on mortality patterns in older age. | | survey | cohort | categorical | no | 10 | None | | mixed | no | no | yes | | age, marital status, education OR occupational status (collinear, so both not included in model), risk of exposure to hazardous agents, smoking, self-reported chronic health conditions, self-rated health, living standard |
| Van Rossum, C.T. et al (2000) | Netherlands | The aim of the study was to describe the relationship between socioeconomic status and mortality in Dutch elderly people. | | survey | cohort | categorical | no | 12 | None | | not significant | no | no | yes | | education, occupation, sex, age, income |
| Vescio, M.F. et al (2003) | Italy | To investigate the association between socio-economic position, overall and cause-specific mortality, and risk factors in a sample of men and women in the Italian population. | | survey | cohort | categorical | no | 14 | None | | not significant | no | no | yes | | sex, year of birth, marital status, occupation, education, smoking, height, weight, BP, EKG, total and HDL, BMI, health status |
| Xie, W. (1996) | China | Using data from the fourth census, this article analyzes the mortality differential for various levels of education in China. | | census | cross sectional | categorical | no | 9 | None | | not significant | no | yes | yes | |  |
| Zajacova, A. (2006) | U.S. | This paper explores gender differences in the education gradient in mortality for US adults. | | survey | cohort | continuous | no | 12 | Pathway | | mixed | no | yes | no | | age, sex, region of birth, urban city, marital status, household size, region of residence, education, income, # of rooms per person in respondents house, smoking, exercise, BMI |
| Zajacova, A. et al (2009) | U.S. | The goal of this paper is to analyze gender differences in education gradients in mortality among non- Hispanic white and black U.S. adults born between 1906 and 1965 | | census | cross sectional | categorical | no | 12 | Pathway | | mixed | yes | no | yes | | age, gender, region of residence, year of interview, marital status, race, education |

**Table S2**: Description of articles in Outcome-Specific Domain, alphabetically. (N=89 Outcome-Specific Domain, N=25 Cancer Sub-Domain)

| Author (yr) | Study Region | Aim | Study Population | Study Design | Education Variable | Conditioned on SES | QR score | Second Domain | Education Effect | Stratified by Race | Stratified by Age | | Stratified by Gender | Predictor Variables |
| --- | --- | --- | --- | --- | --- | --- | --- | --- | --- | --- | --- | --- | --- | --- |
| Abdoli, G. et al (2014) | Sweden | We evaluated the risks of mortality due to all-site cancer and to some common specific cancers in the total Swedish population and in subgroups of the large and currently increasing foreign-born population by individual birth country, sex, and socioeconomic position (SEP). | census | cohort | categorical | no | 14 | Trends | significant | no | no | yes | | Place of birth, sex, age, level of education, marital status |
| Ahacic, K. et al (2012) | Sweden | To determine if socioeconomic position continues to predict mortality, stroke specific, or from other causes, among patients surviving their first stroke in spite of this selective survival. | survey | cohort | categorical | yes | 10 | None | not significant | no | no | no | | age, sex, and stroke type, income, days of inpatient care |
| Ahmadi, A. et al (2014) | Iran | To investigate relationship between risk factors and in-hospital mortality due to MI by educational level. | survey | cohort | categorical | no | 12 | None | not significant | no | yes | yes | | risk factors, years of schooling, age, sex, EL, treatment used, ischemic pain pattern, STMEI/non STEMI |
| Albano, J.D. et al (2007) | United States | We examined relationships among race, education level, and mortality from cancers of the lung, breast, prostate, colon and rectum, and all sites combined in contemporary US vital statistics. | census | cross sectional | categorical | no | 13 | None | significant | yes | no | yes | | race, sex, age, educational attainment, smoking practices, obesity, health insurance, and utilization of mammography and colorectal screening |
| Allen, J.G. et al (2012) | United States | The purpose of this study was to evaluate the effect of recipient insurance and education on survival after orthotropic heart transplantation (OHT). | survey | cohort | dichotomized | no | 14 | None | significant | no | no | no | | educational levels, insurance policies, transplant diagnosis, demographics, pre-OHT mechanical ventilation, and/or ICU admission, comorbidities, and transplant variables, race |
| Álvarez, J.L. et al (2011) | Europe | To describe the magnitude of socioeconomic inequalities in tuberculosis (TB) mortality by level of education in male, female, urban and rural populations in several European countries. | census | cross sectional & cohort | dichotomized | no | 14 | None | significant | no | no | yes | | age, sex, region (urban/ rural, and by country) , education |
| Ariansen, I. et al (2015) | Norway | Our objective was to examine whether repeated measures over time of risk factors (smoking, physical inactivity, blood pressure, total cholesterol and body mass index) explain more of the socioeconomic gradient in CVD mortality than if they are measured only once. | census | cohort | categorical | no | 13 | Pathway | significant | no | no | no | | Education, age, sex, systolic blood pressure, cholesterol, BMI, smoking, physical activity |
| Avendano, M. et al (2004) | Europe | The aim of this report is to assess stroke mortality differences according to educational level across Europe during the 1990s. | census | cohort | dichotomized | no | 11 | None | significant | no | yes | yes | | educational level, sex, and 5-year age group |
| Beauchamp, A. et al (2010) | Australia | We aimed to examine whether a socioeconomic gradient in CVD mortality exists in a large cohort of both men and women, with accurate ascertainment of both endpoints and risk factors, and to describe and quantify the separate effects of behavioural, physiological and social risk factors on the relationship between SES and CVD. | survey | cohort | categorical | no | 13 | Pathway | not significant | no | no | no | | country of birth, age, sex, education, behavioral risks, physiological risk factors |
| Borell, C. et al (1999) | Austria, Belgium, Denmark, Finland, Norway, Switzerland, Spain, Italy | To study the differential distribution of transportation injury mortality by educational level in nine European settings, among people older than 30 years, during the 1990s. | survey | cohort | categorical | no | 13 | None | mixed | no | yes | yes | | age, sex, education, country |
| Bouchardy, C. et al (1993) | Brazil | We report here the relationship between socioeconomic status and cancer mortality in Sao Paulo county in Brazil. | census | cohort | categorical | no | 13 | None | mixed | no | no | yes | | age, education level, civil status, and birthplace |
| Brehaut, J.C. et al (2004) | Canada | The current study will examine the relationship between education and mortality in the light of the spectrum of cognitive decline, ranging from no cognitive impairment (NCI), through CIND, to dementia. | survey | cohort | categorical | no | 13 | None | significant | no | no | no | | age, gender, education, and an initial screening for cognitive impairment |
| Callahan, L. F. et al (1996) | United States | To analyze scores on a scale designed to measure helplessness, a cognitive variable, as a possible mediator of the association between formal education level and mortality over 5 years in patients with rheumatoid arthritis (RA). | other | cohort | dichotomized | no | 12 | None | not significant | no | no | no | | demographic, socioeconomic, therapy, functional status, and psychological variables |
| Chenet, L. et al (2008) | Russia | To examine the association between accidental, violent and alcohol related adult mortality in the Russian capital and socioeconomic status characteristics such as educational status, occupational group and marital status. | census | cross sectional | categorical | yes | 12 | None | significant | no | no | yes | | nationality, date of birth, marital status, occupational group, education, date of death, place of death, cause of death |
| Coady, S.A. et al (2014) | United States | To assess the association of individual education and area income with survival and recurrence post Myocardial Infarction (MI). | census | cohort | categorical | no | 13 | None | not significant | no | yes | yes | | age, sex, race, Hispanic status, educational attainment, marital status, hypertension, hyperlipidemia, diabetes, stroke, CHD, zip code |
| Consuegra-Sanchez, L. et al (2015) | Spain | The aim of this study was to evaluate the impact of educational level, as a marker of socioeconomic status, on the prognosis of long-term survival after acute myocardial infarction. | other | cohort | categorical | no | 12 | None | significant | no | no | no | | education, occupational class, age, sex, diabetes mellitus, history of previous MI, New York Heart Association functional class > 2, stroke, peripheral arterial disease, chronic kidney disease, COPD, neoplasms, atrial fibrillation, HR, and systolic BP, Killip class > I on admission, reperfusion, left ventricular ejection fraction, and time to admission, BMI and active smoking |
| Corey, M.R. et al (2014) | United States | The aim of this study was to examine the relationship between patient education level and 5-year mortality after major lower extremity amputation. | other | cohort | dichotomized | no | 11 | None | significant | no | no | no | | age, race, education, preoperative ambulatory status, history of revascularization attempts, indication for operation, type of operation, average number of postoperative complications, number of amputation revisions, smoking status |
| Cserép, Z. et al (2012) | Hungary | To investigate the relationship between depression, anxiety, education, social isolation and mortality 7.5 years after cardiac surgery. | other | cohort | categorical | no | 12 | None | significant | no | no | no | | MI, Previous CABG, history of arrhythmia, CHF, diabetes mellitus, hypercholesterolemia, cerebrovascular disease, chronic renal insufficiency, hypertension, and history of psychiatric treatment. Age, gender, living status, education, depression, anxiety |
| Dennis, B.H. et al (1993) | Russia | We examined the effect of social status on CHD mortality. | survey | cohort | categorical | no | 9 | None | significant | no | no | n/a | | CHD at baseline, WHO questionnaire on effort angina, angina on exercise testing and use of angina medication, anti-arrhythmic agents, digitalis or propranolol, age, blood cholesterol and triglyceride/ other protein levels, dietary variable, education |
| Donyavi, T. et al (2011) | Iran | This study aimed to investigate whether mortality after myocardial infarction (MI) varies by SES in Iran. | other | cohort | categorical | no | 11 | None | not significant | no | no | no | | age, gender, marital status, employment, BMI, risk factors for MI |
| Dray-Spira, R. et al (2010) | United States | To measure relative and absolute educational disparities in mortality among U.S. adults with diabetes and to compare their magnitude with disparities observed within the nondiabetic population. | census | cohort | categorical | no | 12 | None | mixed | no | no | no | | age, sex, race/ethnicity, diabetes, education |
| Dupre, M.E. et al (2015) | United States | This study investigated the associations among education, hemoglobin A1c (HbA1c), and subsequent mortality in adults with diabetes. | survey | cohort | dichotomized | no | 13 | None | mixed | no | no | no | | HbA1c levels (dichotomized), age, sex, race/ethnicity, time since diagnosis, insulin use, obesity, BP, and non-HDL cholesterol, cardiovascular comorbidity, cystatin C levels, income, insurance, depression, social support, marital status, control over one's health, smoking, alcohol, exercise, doctor visits, occupation, age, BMI, geographic region |
| Egeland, G.M. et al (2002) | Norway | We examined whether wives' education was associated with men's risk of CHD after taking into account the men's own educational level. | survey | cohort | categorical | no | 12 | Pathway | not significant | no | no | n/a | | education, wife's education, diastolic/systolic BP, total serum cholesterol, BMI, smoking habits, sedentary behaviour, BP meds, |
| Elstad, J.I. et al (2012) | Norway | The aim of the present study is to increase knowledge about developments in educational inequalities in mortality from specific cancer sites. | census | cohort | categorical | no | 14 | Trends | mixed | no | no | yes | | sex, age, cancer type, educational level, |
| Ernstsen, L. et al (2010) | Norway | To investigate the influence of psychosocial and behavioural factors on educational inequalities in ischaemic heart disease (IHD) mortality. | survey | cohort | categorical | no | 14 | None | mixed | no | no | yes | | sex, age, SEP, psychosocial factors (marriage status, depressive symptoms, life satisfaction, behavioural factors (alcohol, smoking, physical activity), education |
| Ezendam, N.P. et al (2008) | Poland, Lithuania, Estonia, Finland, Sweden | To compare educational inequalities in cancer mortality between Poland, Lithuania,  Estonia, Finland and Sweden. | census | cross sectional | categorical | no | 12 | None | mixed | no | no | yes | | age, country, cancer site, sex, educational level, |
| Faggiano, F. et al (1995) | Italy | The main objective of this paper is to present the first results of inequalities in cancer mortality in Italy as a whole, based on a nationwide record-linkage between 1981 census data and mortality in the subsequent six months. | census | cohort | categorical | no | 14 | None | mixed | no | no | yes | | age, sex, education, SES, region of country |
| Fedeli, U. et al (2015) | Italy | To investigate the association between education level and mortality from alcoholic, viral, and non-viral/non-alcoholic chronic liver disease. | census | cross sectional | dichotomized | no | 13 | None | mixed | no | no | yes | | gender, age, educational level |
| Feinglass, J. et al (2015) | United States | To analyze the association between SES and all-cause mortality among women diagnosed with breast cancer before and after controlling for insurance status, race and ethnicity, stage, treatment modalities, and other demographic and hospital characteristics. | survey | cohort | categorical | yes | 14 | None | significant | no | no | n/a | | age, race and ethnicity, TNM staging & pathology |
| Fernandez, E. et al (1999) | Spain | The objective of this study was to examine the relationship between educational level and mortality from cancer in the city of Barcelona. | census | cross sectional | categorical | no | 13 | None | mixed | no | yes | yes | | level of education, cancer site, age, gender |
| Gadeyne, S. et al (2012) | Belgium | The analyses will investigate whether educational differences in breast cancer mortality can be explained by reproductive factors. | census | cohort | categorical | no | 13 | Pathway | significant | no | yes | n/a | | age, nulliparity, education, pre/postmenopausal |
| Geerlings, M.I. et al (1997) | Netherlands | The objective of this study was to replicate findings from an earlier study by Stern et al. of an increased risk of mortality in Alzheimer's disease (AD) patients with higher levels of education and to compare this risk with the risk of death in the elderly population. | survey | cohort | dichotomized | no | 12 | None | not significant | no | no | no | | age, sex, MMSE score, CAMDEX score, CAMCOG score, educational attainment |
| Gnavi, R. et al (2004) | Italy | To compare diabetics with non-diabetics in terms of mortality and social differences in mortality, we conducted a study among people with diabetes living in the city of Turin, Italy, and compared them with Turin residents without diabetes. | census | cohort | categorical | no | 11 | None | mixed | no | yes | yes | | type 1 or 2, age, gender, area of birth, education |
| Gnavi, R. et al (2011) | Italy | We investigated if diabetes modifies the effect of the association of education with mortality and incidence of cardiovascular diseases. | census | cohort | categorical | no | 11 | None | mixed | no | no | yes | | gender, age, educational level, treatment (diet only, oral drugs, insulin), mortality/ incidence |
| Gnavi, R. et al (2014) | Italy | In an unselected population of patients admitted to hospital with a first episode of STEMI and NSTEMI we examined gender and socioeconomic differences in the use of cardiac invasive procedures and in one-year mortality. | other | cohort | categorical | yes | 12 | None | not significant | no | no | no | | PCI, PPCI, CABG, In-hospital mortality, gender, age, educational level, Charlson index, admitting ward, angiography facility on-site |
| Goldfarb-Rumyantzev , A. et al (2012) | United States | We tested the hypothesis that higher education might result in reduced disparities in the outcomes of renal transplantation. | survey | cohort | categorical | no | 11 | None | significant | yes | no | no | | race, gender, age (during transplant), educational level, pre-transplant dialysis time, comorbidity index, BMI post-transplant, donor type, cause of ESRD, duration of pre-ESRD nephrology care, type of vascular access, HLA mismatch, and PRA levels |
| Heck, K.E. et al (1997) | United States | To examine individual-level analysis of socioeconomic differences in breast cancer mortality in the US, with education used as the measure of SES. | census | cross sectional | categorical | no | 12 | None | not significant | yes | no | n/a | | age, ethnicity, education, state |
| Herndon, J.E. et al (2013) | United States | This paper aims to investigate the effect of socioeconomic status, as measured by education, on the survival of breast cancer patients treated on 10 studies conducted by the Cancer and Leukemia Group B. | survey | cohort | categorical | no | 14 | None | mixed | no | no | n/a | | African American race, never married, negative estrogen receptor status, >4 significant nodes, tumor diameter >2 cm, and education, age, marital status, menopausal status, ER, PR |
| Holzner, B. et al (2004) | Germany | The aim of the study was to determine the impact of socioeconomic status on relapse-free survival (RFS) in patients with Hodgkin’s disease. | other | cohort | categorical | no | 11 | None | not significant | no | no | no | | Income, Education, different treatment modalities, clinical risk factors (age at diagnosis, stage of disease, involvement of three or more lymph nodes, presence or absence of a large mediastinal mass, E stages or elevation of erythrocyte sedimentation rate (ESR). |
| Huang, W.H. et al (2013) | Taiwan | The aim of this multi-center study was to investigate the relationship between education levels and 3-year mortality rates in HD patients. | other | cohort | dichotomized | no | 11 | None | mixed | no | no | yes | | age, HD duration, hypertension, creatinine level, serum albumin level, anuria, high education, education, CVD, infectious disease, comorbidities |
| Hussain, S.K. et al (2008) | Sweden | To assess the association between education level and in situ and invasive breast cancer risk and invasive breast cancer survival, using the 2006 update of the Swedish Family-Cancer Database. | census | cohort | categorical | no | 14 | None | significant | no | yes | n/a | | age, time-period, parity, age at first birth, county of residence and family history of breast cancer |
| Igland, J. et al (2014) | Norway | The aim of the study was to investigate educational inequalities in mortality among all patients hospitalised for an incident AMI during 2001–2009 in Norway. | survey | cohort | categorical | no | 14 | Trends | mixed | no | yes | no | | level of education, income, Charlson comorbidity Index, age, gender, revascularization techniques (PCI, CABG, PCI/CABG) |
| Islam, M.S. et al (1984) | Bangladesh | To examine differentials in diarrhea episodes, hospitalizations, and mortality rates, according to patients' ages and educational levels, as well as the occupational categories of the heads of households, and dwelling ownership and type. | survey | cohort | categorical | no | 10 | None | not significant | no | yes | no | | educational levels, occupations of the heads of households, dwelling type and ownership, age, gender |
| Jarrin, I. et al (2007) | Spain | To investigate the impact of education on long-term overall and cause-specific mortality, if it has changed over the last decades, and whether any increases in the inequalities in mortality by educational level might be attributed to the introduction of HAART. | survey | cohort | categorical & dichotomized | no | 13 | None | mixed | no | no | no | | education, calendar period interaction, sex, age, HIV, age at first visit, drug use habits (age and year at starting IDU, duration of IDU prior to study entry, and sharing drug-use equipment) |
| Jasilionis, D., et al. (2015) | Lithuania | We investigate relative mortality inequalities by education for detailed cancer sites and provide estimates of deaths which could have been avoided through the elimination of these inequalities. | census | cohort | dichotomized | no | 10 | None | mixed | no | no | yes | | age, gender |
| Kaseliene , S. et al (2011) | Lithuania | The aim of this study was to evaluate changes in inequalities in mortality from infectious diseases and tuberculosis by educational level among men and women in Lithuania. | census | cross sectional | categorical | no | 10 | Trends | significant | no | no | yes | | sex, level of education, census year |
| Kilander, L. et al (2001) | Sweden | We aimed to investigate whether differences in traditional vascular risk factors, adult height, physical activity, and biomarkers of fatty acid and antioxidant intake, could explain this association. | survey | cohort | categorical | no | 12 | None | not significant | no | no | n/a | | educational level, blood pressure, blood glucose, body mass index, serum lipids, smoking, body height, physical activity, serum beta carotene, alpha tocopherol, selenium, and serum fatty acids in cholesterol esters |
| Kim, C. et al (2005) | United States | We examined the association between SES and CVD and breast cancer mortality among black and white women. | census | cohort | categorical | no | 13 | None | mixed | yes | no | n/a | | age, marital status, urban or rural dwelling, education, race |
| Kleefstra, N. et al (2014) | Netherlands | The aim of this study was to estimate relative and absolute educational disparities in mortality in a Dutch cohort of adults with T2DM. | survey | cohort | categorical | no | 10 | None | mixed | no | no | no | | Age, gender, BMI, smoking, macrovascular complications, diabetes duration and working status. |
| Krause, J.S. et al (2009) | United States | To identify the association of social support and socioeconomic factors with risk of early mortality among persons with spinal cord injury. | survey | cohort | continuous | no | 10 | None | not significant | no | no | no | | Behavioral Risk Factor Surveillance System, Reciprocal Social Support Scale, biographic and injury characteristics, including functional injury classification, gender, race, age at the time of injury, and years lived since injury to the time of survey |
| Krishnatreya, M., et al. (2015) | India | The main objective of this paper was to assess the influence of educational level on the survival of uterine cervix cancer patients in our population. | survey | cohort | categorical | no | 8 | None | not significant | no | no | N/A | | stage of cancer, education |
| Kulhanova, I. et al (2014) | Europe | The aim of the present study was therefore to examine whether there are educational differences in the proportion of ill-defined causes of death among men and women in 16 European populations and to investigate if these differences harm the socioeconomic differences in mortality from specific causes of deaths, especially ischemic heart disease or suicide. | survey & census | cross sectional | categorical | no | 13 | None | significant | no | No | yes | | country, sex and education |
| Legarth, R. et al (2014) | Denmark | To estimate association between educational attainment and risk of HIV diagnosis, response to HAART, all-cause, and cause-specific mortality in Denmark in 1998–2009. | survey | cohort | categorical | no | 13 | None | not significant | no | no | no | | demographics, date of HIV diagnosis, route of transmission, AIDS-defining events, ARV treatment, CD4+ cell counts, HIV RNA measurements |
| Lofmark, U. et al (2008) | Sweden | Our aim was to investigate whether there were education-related differences in 28-day case fatality after stroke in different age groups. | other | cohort | dichotomized | no | 12 | None | not significant | no | yes | no | | demographics on admission diagnoses, background characteristics, risk factors for stroke, in-hospital procedures such as acute and prophylactic medication, in-hospital mortality and 28-day case fatality |
| Lund, E. et al (1991) | Norway | We have evaluated the relationship between level of education--as measure of socioeconomic status--and mortality from breast cancer in a large Norwegian prospective study. Further to determine whether established risk factors for breast cancer influence the relationship, we adjusted for parity and age at first birth in a separate set of analyses. | census | cohort | categorical | no | 12 | None | mixed | no | yes | n/a | | number of children, age at first live birth completed years of school, age at marriage |
| Mackenbach, J.P. et al (2004) | England, Wales, Norway, Denmark, Finland, Belgium, Switzerland, Austria, Spain, Italy | The aims of our study were: (1) to describe socioeconomic inequalities in lung cancer mortality in different European populations; (2) to make inferences about the staging of the smoking epidemic in different European populations; (3) to make inferences about the contribution of smoking to socioeconomic inequalities in total mortality in different European populations. | census | cohort | dichotomized | no | 11 | None | significant | no | yes | yes | | cause of death, age, sex, educational level, country |
| Makela P. (1999) | Finland | To describe the differences in acute, chronic and total alcohol-related mortality by several measures of socio-economic status (SES) and to unravel the influences of different dimensions of SES. | survey | cohort | categorical | no | 13 | Trends | significant | no | yes | yes | | education, occupational class, personal income, net household income per consumption unit (spending power) and housing tenure |
| Malyutina, S. et al (2004) | Russia | We examine the association between mortality from all causes and CVD and two markers of socioeconomic status: education and marital status. | survey | cohort | categorical | no | 12 | None | not significant | no | no | yes | | education, marital status, risk factors, sex |
| Manor, O. et al (2004) | Israel | This study examines educational differentials in cardiovascular mortality in Israel for both men and women aged 45 to 69 and 70 to 89 years. | census | cohort | categorical | no | 12 | None | mixed | no | yes | yes | | age at the time of the census, education, possession of a car, ethnic origin |
| Martikainen, P. et al (2000) | Finland | This report examines trends in Finnish breast cancer mortality by education, age, and birth cohort. | census | cohort | dichotomized | no | 13 | Trends | not significant | no | yes | n/a | | education, age |
| Martikainen, P. et al (2001) | Finland | To assess the extent of lung cancer mortality differentials by education while adjusting for exposure to tobacco smoke and asbestos based on survey questions. | survey | cohort | dichotomized | no | 13 | None | not significant | no | no | n/a | | smoking, education, sociodemographic characteristics, age |
| Martin, R.S.S. et al (2012) | Brazil | To extend a previous study and to assess whether left ventricular hypertrophy can explain the association between schooling and cardiovascular mortality in hemodialysis patients. | other | cohort | dichotomized | no | 11 | None | not significant | no | no | no | | education, age, gender, ethnicity, educational level, cause of CKD, monthly family income divided by the number of people in the household, and professional situation |
| McMahon, J. et al (2011) | United States | We investigated whether SES predicts mortality in HIV infected persons in the HAART era using prospective and individually collected data while controlling for possible confounding factors. | survey | cohort | dichotomized | no | 11 | None | not significant | yes | no | yes | | SES measures (hunger, homelessness, poverty, and education), demographics, mode of HIV transmission, harmful behaviors, viral factors, use of HAART, depression, and albumin |
| Mehta, R.H. et al (2011) | UK, US, Germany, Sweden, Italy, NZ, Poland, Canada, Australia | The purpose of this study was to examine the association between lower socioeconomic status (SES), as ascertained by years of education, and outcomes in patients with acute ST-segment elevation myocardial infarction (STEMI) | survey | cohort | categorical & continuous | no | 12 | None | significant | no | no | no | | demographics (age, sex, race, height, weight), medical history (hypertension, diabetes mellitus, current smoking, elevated cholesterol, previous CHF, previous MI, previous CBVD, PCI, CABG), country, Education |
| Mejia-Lancheros, C. et al (2014) | Spain | The present study aims to determine the contribution of psychosocial determinants in increasing the risk of cardiovascular events (myocardial infarction and stroke), and death from CVD, in a high risk adult population. | survey | cohort | categorical & dichotomized | no | 13 | None | not significant | no | no | yes | | education, sex, depression, social support, BMI, smoking, diet, CV risk factors, alcohol |
| Menvielle, G. et al (2006) | France | We investigated the time trends in social inequalities in breast cancer mortality with an analysis by age at death and birth cohort using a representative 1% sample of the French population and four sub cohorts. | census | cohort | categorical & dichotomized | no | 14 | Trends | not significant | no | yes | n/a | | death, birth cohort, education |
| Menvielle, G. et al (2008) | Spain, Slovenia, Italy, Switzerland, France, Belgium, Denmark, Norway, Sweden, Finland | To what extent socioeconomic inequalities in total cancer mortality among women really vary between countries, to which an international comparison of educational disparities in cancer mortality could provide some answers. | census | cohort | categorical | no | 12 | None | mixed | no | no | yes | | education, sex |
| Nilsson, P.M. et al (1998) | Sweden | The purpose of the present study was to examine the relationship between self-reported diabetes mellitus, gender, attained level of education, and socio-economic resources to all-cause mortality risk in a simple random sample of 39 055 subjects, aged 25 to 74 years. | survey | cohort | categorical | no | 12 | None | significant | no | yes | yes | | gender, education, SES, age, housing tenure, marital status, car ownership |
| Nishi, N. (2008) | Japan | The objective of this study was to examine socioeconomic differences in incidence and mortality for all cancer and all of its major sites, that is, stomach, colon/rectum, liver, lung, breast (female only), and prostate. | survey | cohort | categorical | no | 12 | None | not significant | no | no | yes | | birth cohort, BMI, education, smoking, DS02 radiation dose estimates, sex |
| Omsland, T.K. et al (2015) | Norway | Our objective was to investigate educational inequalities in post-hip fracture mortality and to examine whether comorbidity or family composition could explain any association. | survey | cohort | categorical | no | 11 | None | significant | no | no | yes | | sex, education level, marriage status, number of children, charlson morbidity index, age |
| Pednekar, M.S. et al (2011) | India | Influence of education, a marker of SES, on cardiovascular disease (CVD) mortality has not been evaluated in low-income countries. To determine influence of education on CVD mortality a cohort study was performed in India. | survey | cohort | categorical | no | 13 | None | significant | no | no | yes | | age, smoking, BMI, education status, gender, Religion, mother tongue |
| Pincus, T. et al (1985) | United States | The aim of this paper was to identify possible specific prognostic markers in patients with rheumatoid arthritis (RA). | other | cohort | categorical & dichotomized | no | 11 | None | significant | no | yes | no | | age, number of involved hand joints, and three measures of functional capacity, i.e. responses to questions regarding activities of daily living, modified walking, time, and the button test |
| Pincus, T. et al (2007) | United States | To identify possible specific prognostic markers in RA, and determine the relationship between educational attainment and mortality during a 9 year follow up. | other | cohort | categorical | no | 11 | None | significant | no | no | no | | educational level, ADL, anatomic stage, risk factors, age, and other biopsychosocial factors |
| Pratipanawatr, T., et al. (2015) | Thailand | This study was conducted in order to determine the impact of education on mortality due cardiovascular, infectious and renal disease, and cancer among Thai diabetics using data from the Thailand diabetes registry cohort. | other | cohort | dichotomized | no | 13 | None | mixed | no | no | no | | education, demographic data, relevant findings on physical examination, laboratory results during the 12 month period prior to recruitment, medications used (including insulin, oral hypoglycemic agents, anti-hypertensive agents, lipid lowering agents and aspirin) and complications of diabetes |
| Qui, C. et al (2001) | Sweden | To further understand the relationship between education and incidence of clinically diagnosed AD or dementia with mortality data from the Kungsholmen Project. | survey | cohort | categorical & dichotomized | no | 11 | None | mixed | no | no | no | | age, sex, and educational level, global cognitive functioning (MMSE), AD/dementia, occupation (SES) |
| Rahu, K. et al (2009) | Estonia | The aims of this study were to examine socio-demographic differences in alcohol-related mortality in Estonia, and how they changed over time. | census | cross sectional | categorical | no | 10 | Trends | significant | no | yes | yes | | gender, date of birth, date of death, education, ethnicity, marital status, place of residence |
| Rasmussen, J.N. et al (2006) | Denmark | To study how income and educational level influence mortality after acute myocardial infarction. | other | cohort | categorical | no | 13 | None | mixed | no | yes | no | | gross income, age, education, gender, comorbidity (primary and secondary diagnosis) |
| Regidor, E. et al (2002) | Spain | In this study we analyse the relation between educational level and mortality from infectious diseases. | census | cross sectional | categorical | no | 13 | None | significant | no | no | yes | | education, age, employment status, marital status, household size, area of residence based on deprivation category |
| Sanchez-Barriga, J.J. et al (2015) | Mexico | The aim of this study was to determine trends in nationwide mortality rates by state and by socioeconomic region, and to deter-mine the relative risk (RR) of educational level, state of residence and socioeconomic region with PTB mortality from 2000 to 2009. | census | cross sectional | categorical | no | 10 | Trends | significant | no | no | no | | SES (educational level, occupation, health, housing & employment), region, sex |
| Saydah, S. et al (2011) | United States | This study sought to determine how risk of mortality associated with measures of SES among adults with diagnosed diabetes is mitigated by association with demographics, comorbidities, diabetes treatment, psychological distress, or health care access and utilization. | census | cohort | categorical | no | 10 | None | not significant | no | no | no | | education, financial wealth, family income, age, sex, race/ethnicity, and U.S. birth status, BMI, functional limitations |
| Smailyte, G. et al (2012) | Lithuania | The aim of this study is to describe associations between incidence and mortality by major cancer sites and education in Lithuania. | census | cross sectional | categorical | no | 13 | None | mixed | no | no | yes | | education, cancer type, age, sex |
| Sobrino-Vegas, P. et al (2012) | Spain | The aim of this study was to analyse associations between educational level and delayed HIV diagnosis (DD), late initiation of combined antiretroviral therapy (cART), overall and in subjects with timely HIV diagnosis, virological and immunological responses to cART, and mortality from HIV diagnosis and cART initiation. | survey | cohort | categorical | no | 13 | None | significant | no | no | yes | | education, CD4 count, response to tx, |
| Stern, Y. et al (1995) | United States | To investigate rates of mortality in patients with pAD as a function of educational and occupational attainment (EOA). | survey | cohort | dichotomized | yes | 10 | None | significant | no | no | no | | clinical dementia rating, education, occupation. Age, gender |
| Strand, B. H. et al (2014) | Norway | In this study we investigated the relationship between educational level and dementia related deaths for cohorts of people all born during 1915–39. | survey | cohort | categorical | no | 12 | Pathway | significant | no | yes | no | | educational attainment, age, sex, history of cardiovascular disease, smoking, physical inactivity, adiposity, high cholesterol level and elevated blood pressure |
| Strand, B.H. et al (2007) | Nordic Countries, England, Wales, Belgium, France, Austria, Switzerland, Italy, Spain | We looked specifically at variations between countries in educational inequalities in breast cancer mortality. | census | cohort | categorical | no | 13 | None | mixed | no | yes | n/a | | education, marital status, gender, age, duration of marriage, nationality, type of household, urbanisation, religion, |
| Vandenheede, H. et al (2015) | Nordic countries, UK, Baltic region, Europe | To evaluate educational inequalities in diabetes mortality in Europe in the 2000s, and to assess whether these inequalities differ between genders. | survey & census | cross sectional | categorical | no | 11 | None | significant | no | no | yes | | education, gender, age, country |
| Wagener, D.K. et al (2015) | United States | Hispanics, particularly Mexican Americans, are known to have a higher incidence of mortalities whose underlying cause is a gallbladder-related disorder. These analyses evaluate the role of educational attainment in the differential mortality experiences of these populations. | census | cross sectional | categorical & dichotomized | no | 9 | None | significant | yes | no | yes | | race/ethnicity, educational attainment |
| Ward, M.M. (2004) | United States | I examined the hypothesis that socioeconomic status, measured by education level, was inversely associated with mortality due to SLE in whites, African Americans, and Asian/Pacific Islanders in the United States, using national data on deaths from 1994 to 1997. | census | cross sectional | categorical | no | 13 | None | significant | yes | no | yes | | age, race, yrs of education, place of death |
| Woodward, M. et al (2015) | China, Hong Kong, Japan, Singapore, Taiwan, Thailand, Australasia | A direct comparison of the effects of the same measure of SES between Oriental and Occidental populations. | survey | cohort | categorical | no | 12 | None | not significant | no | no | no | | age, years of follow-up, recorded date of birth, sex, and blood pressure at baseline and date of, or age at, death during follow up, educational attainment, alcohol status, BMI, smoking status, SBP, blood cholesterol, diabetes, event status |

**Table S3**: Description of articles included in Trends Over Time Domain, alphabetically. (N=54)

| Author (yr) | Study Region | Aim | Study Population | Study Design | Education Variable | Conditioned on SES | Quality Ranking Score | Second Category | Education Effect | Stratified by Race | Stratified by Age | Stratified by Gender | Predictor Variables |
| --- | --- | --- | --- | --- | --- | --- | --- | --- | --- | --- | --- | --- | --- |

| Chen, A.Y. et al (2011) | United States | To describe trends in mortality rates for patients with oral cavity and pharynx cancer by educational attainment, race/ethnicity, sex, and association with human papillomavirus infection. | census | cohort | categorical | no | 8 | Cancer | not significant | yes | no | yes | state, race, ethnicity, sex, and educational attainment, HPV association |
| --- | --- | --- | --- | --- | --- | --- | --- | --- | --- | --- | --- | --- | --- |
| Cokkinides, VE et al (2012) | United States | To evaluate overall trends in melanoma mortality rates among non-Hispanic whites by educational level. | census | cohort | categorical | no | 12 | None | not significant | no | no | yes | sex, education, age, race |
| De Grande, H. et al (2013) | Belgium | To gain insight into differences in mortality according to own educational level and how these differences may change over time. | census | cohort | categorical | no | 12 | None | not significant | no | no | yes | educational level, sex, age |
| De Grande, H. et al (2014) | Belgium | This study charts temporal trends in young-adult mortality by urbanization degree between the 1990s and the 2000s in Belgium, taking account of educational level. | census | cohort | categorical | no | 12 | None | not significant | no | no | yes | sex, degree of urbanization, educational level |
| De Grande, H. et al (2015) | Belgium | This study addresses educational inequalities in young-adult mortality between the 1990s and the 2000s by comparing trends in the three different regions in Belgium stratified by sex. | census | cohort | categorical | no | 11 | None | mixed | yes | no | yes | educational level, sex, period, region, employment |
| de Vries, E. et al (2015) | Colombia | We evaluate differences in cancer mortality by educational level and assess time trends in mortality from the most important cancer sites distinguishing infection related cancers and frequently occurring cancer types associated with other risk factors. | census | Cohort | categorical | no | 14 | Cancer | significant | no | no | yes | sex, educational level, age, region, rural/urban, marital status, age |
| de Vries, E., et al. (2016) | Colombia | To evaluate trends in premature cancer mortality in Colombia by educational level in three periods: 1998-2002 with low healthcare insurance coverage, 2003-2007 with rapidly increasing coverage and finally 2008-2012 with almost universal coverage (2008-2012). | survey | cross sectional | categorical | no | 11 | Cancer | significant | no | yes | yes | age, birth cohort, gender, education |
| Doblhammer, G. et al (2015) | Austria | This is the first study documenting trends in mortality differentials of Austrian men and women. | census | cross sectional | categorical | no | 13 | None | significant | no | yes | yes | sex, age, education, occupation |
| Elstad, J. I. et al (2015) | Norway | The article investigates the impact of immigration on educational mortality differences among adults in Norway. | census | cohort | categorical & dichotomized | no | 12 | None | significant | no | no | yes | immigrant status, sex, age, cohort, educational attainment |
| Fawcett, J et al (2005) | New Zealand | This paper explores whether inequalities in mortality by education were greater, and increased more, in New Zealand than in Nordic countries, and determines the contribution of CVD to these differences and trends. | census | cohort | categorical | no | 10 | None | significant | no | no | yes | country, sex, 5-year age groups, level of education |
| Feldman, JJ et al (2005) | United States | The aim is to suggest reasons for mortality trends, to help to target specific subpopulations for allocation of health resources, and to suggest etiologic factors for specific causes of death. | survey | cross sectional & cohort | categorical & dichotomized | no | 11 | None | significant | no | yes | yes | education, age, cigarette smoking, systolic blood pressure, BMI, serum cholesterol, sex |
| Flanagan, L. et al (2015) | England and Wales | This study explored the potential to use the Longitudinal Study to describe trends in mortality inequality by educational attainment in England and Wales from 1971 to 2009 and the limitations in the available data. | census | cohort | categorical | no | 13 | None | not significant | no | no | yes | age, education, occupation |
| Goldring, T., et al. (2016) | United States | We develop a flexible test for changes in the SES-mortality gradient that accounts for changes in the distribution of education, the most commonly used marker of SES. | survey | cohort | categorical | yes | 11 | None | mixed | yes | no | yes | gender, birth cohort, education, race |
| Hadden, W et al (2008) | United States | To assess the trend in inequality in mortality by educational attainment in 2000 and compare it with rates from 1960 and 1986, using relative and absolute indexes of inequality. | census | cross sectional | categorical | no | 9 | None | not significant | yes | yes | yes | age, sex, ethnicity, education, state-level measures of ethnic composition, education, income, income inequality, and region |
| Hayward, M et al (2014) | United States | We draw on this review and our updated evidence to reflect on the question whether education's association with adult mortality has become increasingly causal in recent decades, among whom, why, and the potential research, policy, and global implications of these changes. | census | cross sectional | categorical | no | 10 | Predictor | significant | yes | yes | yes | sex, race, and educational attainment |
| Jaffe, D et al (2008) | Israel | The goals of the present study were to assess changes in educational differentials in overall and CVD mortality over time among Israeli Jews, and explore how such changes may have been affected by the dynamic nature of the population. | census | cohort | categorical | no | 13 | None | significant | no | yes | yes | age, country of origin, education, car ownership |
| Jemal, A. et al (2008) | United States | We examined mortality trends for all-causes and seven leading causes of death in relation to educational attainment from 1993 through 2001. | census | cross sectional | categorical & dichotomized | no | 12 | None | mixed | yes | no | yes | race/ethnicity, education, gender |
| Jemal, A. et al (2013) | United States | Mortality rates continue to increase for liver, esophagus, and pancreatic cancers in non-Hispanic whites and for liver cancer in non-Hispanic blacks. The aim of this paper is to measure the extent these trends vary by socioeconomic status. | census | cross sectional | categorical | no | 9 | Cancer | mixed | yes | no | yes | educational attainment, state, age, race |
| Kalediene, R et al (2005) | Lithuania | The aim of this study was to examine the changes in mortality differentials by level of education during the period of socio-economic transition in Lithuania. | census | cross sectional | categorical | no | 8 | None | mixed | no | yes | yes | education, gender, place of residence, age |
| Kalediene, R et al (2006) | Lithuania | To assess inequalities in mortality from external causes by the level of education and the place of residence during the period of socio-economic transition in Lithuania. | census | cross sectional | categorical | no | 9 | None | not significant | no | no | yes | place of residence, level of education, age, sex |
| Kim, M.H. et al (2015) | Korea | We evaluated the influence of SES on female cancer mortality by examining time trends of socioeconomic inequality according to educational level over the last decade in Korea. | census | cross sectional | categorical | no | 11 | Trends | mixed | no | yes | n/a | age, sex, educational status, date of death, occupation, social class, income, wealth, marital status, race, social relationship indices |
| Kinsey, T. et al (2008) | United States | Population study in which death certificates covering 86% of US deaths in 1993 – 2001 were analyzed for cause of death and level of education of the decedent. | census | cross sectional | categorical | no | 11 | Cancer | not significant | yes | no | yes | age, sex, race, cancer site, state, educational attainment |
| Kolodziej, H.et al (2007) | Poland | We examined changes in education-specific rates of premature mortality among urban adults in Poland during the 1990s, the period of the country’s rapid transition from socialist to free market economy. | census | cross sectional | categorical | no | 13 | None | significant | no | yes | yes | age, sex , education-specific rate of mortality |
| Kravdal, H. (2014) | Norway | To analyze changes in the effects of educational level with respect to cancer survival, which has not been done before? | census | cohort | categorical | no | 11 | Cancer | significant | no | yes | yes | age, sex, education level, type of cancer, marital status |
| Lee, W et al (2009) | Korea | This study aims to examine how inequalities in suicide by education changed during and after macroeconomic restructuring following the economic crisis of 1997 in South Korea. | census | cross sectional | categorical | no | 14 | None | significant | no | yes | yes | age, gender, residential areas (city, county, ward), and education |
| Leinsalu, M et al (2003) | Estonia | We aimed to analyze mortality changes by education from 1989 to 2000 in order to assess the impact of recent changes in Estonia, as well as the delayed effects of pre-transitional developments. | census | cohort | categorical | no | 12 | None | significant | no | yes | yes | age, sex, gender |
| Leinsalu, M et al (2009) | Poland, Estonia, Hungary, and Lithuania | The aim of this study is to assess changes in educational inequalities in total and cause-specific mortality in four Eastern European countries between 1990 and 2000. | census | cross sectional | categorical | no | 14 | None | significant | no | no | yes | age, year, sex, educational attainment |
| Lim, D. et al (2015) | South Korea | The purpose of this study was to examine the change in the PAF of lower educational levels for mortality in Korea, where educational attainment has improved and is associated with the exacerbation of inequalities in mortality levels. | census | cross sectional | categorical | no | 14 | Predictor | significant | no | yes | yes | age, education, sex |
| Ma, J et al (2012) | United States | In this study, we updated the data for 26 states included in our previous analysis by six additional data years (1993–2007) to present contemporary patterns of educational disparities in mortality rates from all causes and five major causes (cancer, heart disease, stroke, diabetes, and accidents). | census | cross sectional | categorical | no | 9 | None | mixed | no | no | yes | educational attainment, state, age, race |
| Martikainen, P et al (2007) | Finland | To estimate changes in the total and independent effects of education and occupational social class on mortality over 30 years, and to assess the causes of changes in the independent effects. | census | cohort | categorical | yes | 11 | Predictor | not significant | no | yes | yes | education, occupational social class, age, sex |
| Menvielle, G. et al (2013) | France | The aim of this study is to estimate relative and absolute educational differences in cancer mortality in France between 1999 and 2007, and to compare these inequalities with those reported during the 1990s. | census | cohort | categorical | no | 14 | Cancer | mixed | no | no | yes | education level, age, sex |
| Moe, J et al (2012) | Norway | We investigated trends in relative risk (rate ratios) and absolute risk (rate differences) of educational inequalities in old age mortality in Norway in the period 1961 to 2009 during which considerable changes in mortality, health policy, and expansion of a comprehensive welfare state occurred. | census | cohort | dichotomized | no | 11 | None | mixed | no | yes | yes | educational level, gender |
| Montez, J et al (2011) | United States | To provide new evidence on trends in the education-mortality gradient from 1986 through 2006 by race, gender, and age among non-Hispanic Whites and Blacks using data from the 2010 release of the National Health Interview Survey Linked Mortality File. | census | cohort | categorical | no | 11 | None | mixed | yes | yes | yes | age, gender, race, education, occupation |
| Montez, J et al (2013) | United States | We investigated three explanations—social psychological factors, economic circumstances, and health behaviors—for the widening education gap in mortality from 1997 to 2006 among white women aged 45 to 84 years using data from the National Health Interview Survey Linked Mortality File. | census | cohort | categorical | no | 13 | None | mixed | no | no | n/a | education levels, age |
| Montez, J et al (2013) | United States | To elucidate why the inverse association between education level and mortality risk (the gradient) has increased markedly among White women since the mid-1980s, we identified causes of death for which the gradient increased. | census | cohort | categorical & dichotomized | no | 12 | None | significant | no | no | n/a | marriage, psychological distress, spouse's education, smoking, obesity, age, education |
| Montez, J et al (2014) | United States | We investigated trends in the educational gradient of US adult mortality, which has increased at the national level since the mid-1980s, within US regions. | census | cohort | categorical | no | 12 | None | significant | no | no | yes | race, gender, age, education level, region |
| Ostergren, O. (2015) | Sweden | The aim of this study is to disentangle the role of income and family type in educational inequalities in mortality in Sweden during 1990–2009, focusing on gender differences. | census | cross sectional | categorical | no | 11 | Pathway | significant | no | no | yes | sex, education level, age, income, family type, person-months at risk, |
| Pappas, G. et al (1993) | United States | This study examines changes in mortality rates from 1960 through 1986 according to income and level of education among persons 25 to 64 years of age in the United States. | census | cross sectional | categorical & dichotomized | no | 9 | None | significant | yes | no | yes | sex, education level, race, family status, income |
| Puigpinos, R. et al (2009) | Barcelona | The objective of this study was to assess trends in cancer mortality by educational level in Barcelona from 1992 to 2003. | census | cohort | categorical | no | 12 | Cancer | mixed | no | no | yes | educational level, age, sex |
| Rau, R. et al (2008) | Austria | To investigate social inequality in mortality after AMI on a large scale (nationwide data) using individual level information on both education and income to study the interrelations between these two indicators. | census | cross sectional | categorical & dichotomized | no | 13 | None | significant | no | no | yes | sex, educational level, census date, |
| Reither, E. N. et al (2006) | Wisconsin | Our study updates this body of knowledge by using recent death certificate and census data from the state of Wisconsin to examine changes in educational disparities in premature mortality over the period 1990-2000. | census | cross sectional | categorical | no | 10 | None | significant | no | yes | yes | education, age, race, gender |
| Richardson, R. et al (2015) | United States | We estimated trends in drug poisoning death rates by educational attainment and investigated educational inequalities in drug poisoning mortality by race, gender, and region. | census | cross sectional | categorical | no | 12 | Outcome Specific | significant | yes | no | yes | gender, race, region, and educational attainment, age, state of residence |
| Rognerud, M. A. et al (2006) | Norway | The aim of this paper was to measure socio-economic inequalities in mortality over a 27-year period and to estimate the simultaneous effects of education and income adjusted for changing proportions and potential confounders. | census | cross sectional | categorical | no | 13 | None | significant | no | no | yes | residential area, education, income, household size |
| Schwarz, F. et al (2008) | Austria | In Austria, educational differentials in all-cause mortality increased in the decade between 1981/82 and 1991/92. The aim of this study was to identify which causes of death contributed most to this increase. | census | cross sectional | categorical | no | 13 | None | significant | no | no | yes | sex, education, census year |
| Shkolnikov, V. M. et al (1998) | Russia | To describe mortality differences by education around the 1979 census and compare these to differentials around the 1989 census. Secondly, to use information from the 1994 micro-census to provide a more provisional picture of the way in which the morality crisis of the 1990s had affected different educational groups. | census | cross sectional | categorical & dichotomized | no | 10 | None | not significant | no | yes | yes | sex, age, educational level, cause of death |
| Shkolnikov, V. M. et al (2012) | Finland, Sweden, Norway | Using high quality, census-linked data and sensible inequality measures, this study documents the changes in absolute and relative mortality differences by education in Finland, Norway and Sweden over the period 1971 to 2000. | census | cross sectional | categorical | no | 11 | None | not significant | no | no | yes | sex, educational group, country of origin |
| Simard, E. P. et al (2012) | United States | We examine trends in HIV mortality by individual levels of educational attainment (as a proxy for SES) and by sex and race/ethnicity for the major segments of the population affected by the HIV epidemic. | census | cross sectional | categorical | no | 11 | Outcome Specific | mixed | yes | no | yes | education, race, age, gender |
| Simard, E.P. et al (2011) | United States | Herein, we examine recent trends in cervical cancer mortality among non-Hispanic whites, non-Hispanic blacks, and Hispanics using individual-level education as a marker of SES as well as trends in the risk of late-stage disease at diagnosis by race/ethnicity and insurance status. | census | cohort | categorical | no | 9 | Cancer | mixed | yes | no | n/a | education level, race/ethnicity, age, state, insurance status, risk status, and histology |
| Strand, B. H. et al (2010) | Norway | We determined how much educational inequalities in mortality widened in Norway during 1960-2000 and which causes of death were the main drivers of this disparity. | census | cohort | categorical | no | 11 | None | mixed | no | no | yes | education, gender, causes of death, death cohort |
| Strand, B. H. et al (2014) | Norway | Using national cause specific mortality data, we build on our previous work to examine if the educational inequalities in mortality in Norway have continued to increase after the millennium and which causes are important regarding the educational inequalities. | census | cross sectional | categorical | no | 13 | None | significant | no | no | yes | education, age, gender |
| Stringhini, S. et al (2015) | Italy | In this study, we aim to examine long-term trends in absolute and relative educational inequalities in overall and cause-specific mortality in Italy. | census | cross sectional | categorical | no | 11 | None | significant | no | no | yes | education, gender, age, |
| Torssander, J., et al. (2016) | Sweden | Using administrative population registers from 1971 and onwards, education-specific annual changes in the risk of death and hospital admission were estimated with complimentary log-log models. | census | cross sectional | categorical | no | 14 | None | significant | no | yes | yes | birth year, education, sex, first hospital admission |
| Wagenaar, K. P. (2015) | France | The aim of this study was to compare the educational, differences in lung and UADT cancer mortality in France for the, periods from 1990 to 1998 and 1999 to 2007. | census | cohort | categorical | no | 13 | Cancer | significant | no | no | yes | education |
| Wamala, S. et al (2006) | Sweden and New Zealand | The aim of this present study was to analyze trends in absolute socioeconomic inequalities in mortality in Sweden and New Zealand. | census | cohort | categorical | no | 12 | None | mixed | no | no | yes | education, income, age, gender, and family type |

**Table S4**: Description of articles included in Explanatory Pathway Domain, alphabetically.

| Author (yr) | Study Region | Aim | Study Population | Study Design | Education Variable | Conditioned on SES | Quality Ranking Score | Second Category | Education Effect | Stratified by Race | Stratified by Age | Stratified by Gender | Predictor Variables |
| --- | --- | --- | --- | --- | --- | --- | --- | --- | --- | --- | --- | --- | --- |

| Amaducci, L. et al (1998) | Italy | The objective of this study was to assess the association between education, disability, and mortality. | survey | cohort | categorical & dichotomized | no | 12 | None | not significant | no | no | yes | Education, level of disability, sex, occupation, smoking, activity of daily living, alcohol habit, ischemic heart disease, arrhythmia, angina, peripheral arterial disease, hypertension, peripheral neuropathy of lower limbs, arthritis, number of diseases. |
| --- | --- | --- | --- | --- | --- | --- | --- | --- | --- | --- | --- | --- | --- |
| Behrman, J.R. et al (2011) | Denmark | This study’s principal goals are to describe the associations between schooling and (a) hospitalization in 1980–2002 and (b) mortality by 2003 for the 1921–1950 birth cohorts in Denmark, and to investigate for the same cohorts the causal impact of schooling on health and mortality, net of endowments, using rich data on twins from the Danish Twin Registry that are linked to Danish population-level registries. | survey | cohort | categorical | no | 12 | Predictor | not significant | no | no | no | gender, age, educational attainment, MZ/DZ pairs |
| Blakely, T. et al (2005) | New Zealand | The contributions of tobacco smoking to overall mortality and socioeconomic inequalities in mortality vary between populations and over time. We determined how these contributions varied by sex and over time in two national New Zealand cohort studies. | census | cohort | dichotomized | no | 13 | Trends | significant | no | no | yes | smoking, age, sex, ethnicity, education |
| Bosma, H. et al (1995) | Netherlands, Lithuania | To investigate the extent to which the impact of the wife's SES on her husband's risk of premature mortality or CHD is independent of his own SES in two countries with different socioeconomic systems. | survey | cohort | categorical | no | 11 | None | mixed | no | no | n/a | education, education of spouse, risk factors, age, city |
| Charafeddine, R. et al (2012) | Belgium | Belgian Health Interview Surveys of 1997 & 2001, aged 40yrs or older | survey | cohort | categorical | no | 13 | None | mixed | no | no | yes | smoking, age, sex, SES |
| Clay, C.M. et al (1988) | United States | Chicago Heart Association Detemction Project in Industry (CHA), Chicago Western Electric Company Study (WE) , Chicago Peoples Gas Company Study (PG). Men of varying ages above 40yrs | survey | cross sectional & cohort | categorical | no | 11 | None | mixed | no | no | no | employment agency, education, smoking |
| Dalen, J.D. et al (2012) | Norway | The aim of this study was to test whether the association between self-rated health and mortality differs between educational groups in Norway, and to examine whether health problems and health-related behaviour can explain any of these differences within a previously unexplored contextual setting. | survey | cohort | categorical | no | 11 | None | not significant | no | no | yes | education, SRH, marital status, health related variables, age, sex |
| Davey Smith, G. et al (1998) | Scotland | The objective of this analysis is to demonstrate the profile of mortality differentials, and the factors underlying these differentials, which are associated with the two socioeconomic measures. | survey | cohort | categorical | yes | 14 | None | not significant | no | yes | no | age, age at which the participant finished full time education, occupational social class, whether a regular car driver, health status measures, health related behaviours, physical examination |
| De Grande, H., et al. (2015) | Belgium | This study addresses the following research question: Is educational intergenerational mobility associated with all-cause and cause-specific mortality in young adulthood? | census | cohort | categorical | no | 12 | None | mixed | no | no | yes | age, parental education, parental employment status, personal education, personal employment, personal housing status, parental housing status, longstanding illness, gender |
| Denney, J.T. et al (2010) | United States | This paper thus takes a contingent approach to examining how smoking mediates the relationship between education and mortality. | census | cross sectional | categorical | no | 11 | None | mixed | no | yes | no | age, gender, race/ethnicity, marital status, employment, income, smoking, education |
| Federico, B. et al (2013) | Italy | In this study, we aimed to assess the size of mortality inequalities in Italy as a whole, their geographical pattern of variation within Italy, and the contribution of smoking to these inequalities. | census | cohort | categorical | no | 12 | None | mixed | no | no | yes | sex, age, education, region, smoking status |
| Herttua, K. et al (2015) | Finland | Our aim was to determine, by means of time-series analysis, the association of minimum prices for alcohol, overall and for different types of alcoholic beverages, with alcohol-related mortality among men and women across three educational groups. | census | cohort | categorical | no | 11 | None | not significant | no | no | yes | education, gender, price of alcohol |
| Huisman, M. et al (2007) | Netherlands | The purpose of this study was to assess potential differences in the predictive ability of self-assessed health for mortality between educational groups, and to find explanations for any of these educational differences. | survey | cohort | categorical | no | 13 | None | mixed | no | no | yes | self rated health, health behavior (smoking, alcohol), big life events, education, marital status |
| Jaffe, D.H. et al (2005) | Israel | To examine educational gradients in overall and cause-specific mortality among elderly married men and women and their spouses. | census | cohort | categorical | no | 14 | None | not significant | no | no | yes | continent of birth, age, sex, education level, spouse's educational level |
| Jaffe, D.H. et al (2006) | Israel | Specific goals were (a) to assess the contribution of a spouse’s educational attainment on one’s overall and CVD mortality and (b) to determine if educational discrepancy between spouses affects mortality. | census | cohort | categorical | no | 14 | None | significant | no | no | yes | age, country of birth, continent of origin, education, education gap between spouses, car possession , sex |
| Khang, Y.H. et al (2009) | South Korea | This study examined relative and absolute abilities of material, psychosocial, and behavioral pathways to explain educational and occupational inequalities in mortality in a nationally representative sample from South Korea. | survey | cohort | categorical | no | 11 | None | significant | no | no | no | material factors (income, health insurance, car ownership), psychosocial factors (depression, stress, and marital status), behavioral factors (smoking, alcohol consumption, phsyical exercies), age, sex, education, and occupation |
| Kopp, M.S. et al (2005) | Hungary | To examine the relations between subjective social status, and objective socioeconomic status (as measured by income and education) in relation to male/female middle aged mortality rates across 150 sub-regions in Hungary. | census | cross sectional | categorical | yes | 11 | None | significant | no | no | yes | sex, age, subregion, subjective social status, personal income, education |
| Kravdal, O. (2008) | Norway | The objective of this study was to find out whether the educational achievements of family members and people in the municipality have an impact on a person’s mortality, net of the well-known strong influence of his or her own education. | census | cohort | categorical | no | 12 | None | significant | no | no | yes | education (self & others), marriage status, age, sex |
| Kulik, M.C. et al (2014) | Europe | The aim of this study was to provide estimates of educational inequalities in mortality from three smoking-related causes, for those 30–74 years old, for the early 2000s in 18 European populations, and to also compare the inequality patterns between the different CoD. | census | cross sectional & cohort | categorical | no | 10 | None | mixed | no | yes | yes | age, sex, education, smoking related diseases, lung cancer |
| Laaksonen, M. et al (2008) | Finland | The aim of this study was to examine the effect of health behaviours on relative differences in cardiovascular and all-cause mortality by educational level. | survey | cohort | categorical | no | 11 | None | mixed | no | no | yes | education, sex, behavioral tendencies: smoking status, alcohol use, leisure time physical activity, vegetable use, saturated fat in daily diet, coffee drinking, BMI |
| Lager, A. et al (2009) | Sweden | To establish whether differences in early IQ explain why people with longer education live longer, or whether differences in father’s or own educational attainment explain why people with higher early IQ live longer. | survey | cohort | categorical | no | 10 | None | mixed | no | no | yes | year of birth, gender, father's education, own education, educational attainment, IQ |
| Lager, A. et al (2012) | Sweden | In this study, we assess the hypothesis that individual change in the performance on IQ tests between ages 10 and 20 years is associated with mortality later in life. | survey | cohort | continuous | no | 11 | None | not significant | no | no | no | intelligence scores at ages 10 and 20 years, years of schooling between these ages, and paternal education |
| Liao, C.C. et al (2014) | Taiwan | To evaluate whether the effects of providing or receiving social support are more beneficial to reduce mortality risk among the elderly with different educational levels. | survey | cohort | dichotomized | no | 11 | None | mixed | no | no | no | average age, sex, median survival years within the study period, education, income level, major diseases, the 10-item CES-D, the 5-item Short Portable Mental Status Questionnaire, ADL, IADL, self rated health, receiving and providing social support |
| Lusyne, P. et al (2001) | Belgium | The aim of this paper is to ascertain whether and to what degree the mortality-increasing effect of bereavement differs with respect to the time since bereavement, sex, age, and, in particular, educational level. | census | cohort | categorical | no | 14 | None | not significant | no | no | no | age/time since bereavement, sex, and age, education |
| Mackenbach, J. et al (2015) | Europe | We compared the magnitude of inequalities in mortality between more and less preventable causes of death in 19 European populations, and assessed whether inequalities in mortality from preventable causes are larger in countries with larger resource inequalities. | census | cross sectional & cohort | categorical & dichotomized | no | 10 | None | mixed | no | no | yes | education, sex, age, smoking, alcohol abuse, overweight, low fruit and vegetable intake, physical inactivity and unsafe sex, country of origin |
| Madsen, M. et al (2010) | Denmark | To disentangle an independent effect of educational status on mortality risk from direct and indirect selection mechanisms, the authors used a discordant twin pair design, which allowed them to isolate the effect of education by means of adjustment for genetic and environmental confounding per design. | survey | cohort | categorical & dichotomized | no | 13 | Predictor | not significant | no | no | yes | MZ/DZ, education, birth cohort, gender |
| Martikainen, P. et al (1998) | Finland | We estimate the effects of the death of a spouse on the mortality of the survivor in different education and income groups. | census | cohort | dichotomized | no | 12 | None | mixed | no | yes | yes | sex, age group, education, study year, widowed status |
| Masters, R.K. et al (2015) | United States | In the present study, we explore the existence of a fourth “fact” concerning FCT by testing whether the education gradient in US adult mortality risk grew more rapidly for more preventable causes of death than did the gradient in mortality risk from less preventable causes during a time of significant reductions in US adult mortality. | census | cross sectional | categorical | no | 13 | None | mixed | yes | no | yes | race, age, sex, education, preventability |
| McFadden, E. et al (2008) | United Kingdom | To investigate the independent associations between occupational and educational based measures of socioeconomic status (SES) and cause-specific mortality, and the extent to which potentially modifiable risk factors smoking and body mass index (BMI) explain such relationships. | survey | cohort | categorical | no | 13 | Predictor | mixed | no | no | yes | smoking, BMI, education, social class, gender, age |
| Montez, J. (2015) | United States | In this study, we provide new evidence about the reasons for the widening gap in all-cause mortality risk across education levels among white women. | census | cross sectional | categorical | yes | 13 | None | mixed | yes | no | yes | education, employment, sex, marital status, family income, smoking status, obesity status, race, age |
| Naess, O. et al (2012) | Norway | To estimate the impact family factors shared by siblings has on the association between length of education and cause-specific mortality in adulthood. | census | cohort | categorical | no | 13 | None | significant | no | yes | yes | education, age, sex, mortality, number of siblings |
| Nordahl, H. et al (2014) | Denmark | We aimed to quantify how much of the educational inequality in cause-specific mortality was mediated by four behavioral risk factors. We also aimed to separate the effects of differential exposure and differential vulnerability to these behavioral risk factors simultaneously. | survey | cohort | categorical | no | 13 | Predictor | mixed | no | no | yes | education, smoking, alcohol intake per week, physical activity, BMI (all of these were combined on a 16 level scale), age, sex, cohort |
| O'Neill, M.S. et al (2008) | Mexico, Chile, Brazil | We examined whether educational level modified associations between mortality and ambient particulate pollution (PM10) in Latin America, using several timescales. | census | cross sectional | categorical | no | 13 | None | not significant | no | no | yes | age, sex, educational attainment, natural spline for temporal trend, linear PM10 and apparent temperature at matching lags, and day-of-week indicators |
| Regidor, E. et al (2015) | Spain | The present study aims to add to the empirical evidence by showing mathematically and graphically the relation of latitude and longitude to total mortality and to mortality from the leading causes of death in groups with lower and higher education in Spain, as well as the geographic pattern of mortality inequalities by education. | census | cohort | dichotomized | no | 12 | None | mixed | no | no | no | Latitude, Longitude, education, rurality, age,sex |
| Reques, L. et al (2015) | Spain | This study aimed to investigate the relationship between education and different indicators of material wealth with mortality, and to analyze whether this relationship varies with the leading causes of death. | census | cohort | categorical | no | 13 | None | significant | no | yes | yes | education, number of rooms in home, surface area of home and number of vehicles, age, sex |
| Rogers, R.G. et al (2013) | United States | To determine to what extent sets of economic and social resources, health behaviors, and physiological measures independently mediate the relationship between educational attainment and all-cause adult mortality and to what extent these mediating factors are moderated by age. | survey | cohort | categorical | no | 13 | None | mixed | yes | yes | no | educational attainment, age, sex, race, family income, marital status, visits with friends/relatives, club involvement, health behaviors, physiological indicators |
| Ross, C.E. et al (2012) | United States | To answer “Do women depend more on education for their health and survival than men?” | census | cross sectional | categorical | no | 13 | None | significant | yes | no | no | education, gender, self reported health, age, year of birth, race/ethnicity, proxy respondent |
| Schnohr, C. et al (2004) | Denmark | The aim of the present study is to examine whether the influence on mortality of four health-related behaviours differs by social class. | survey | cohort | categorical | no | 11 | None | significant | no | no | yes | education, smoking, etoh, physical activity, BMI |
| Schrijvers, C.T. et al (1999) | Netherlands | To determine the role of behavioral and material factors in explaining educational differences in all-cause mortality, taking into account the overlap between both types of factors. | survey | cohort | categorical | no | 13 | None | mixed | no | no | no | education, age, gender, marital status, degree of urbanization, religious affiliation, chronic illnesses, etoh consumption, smoking, BMI, physical activity, dietary habits, financial problems, adverse neighborhood conditions, adverse houseing conditions, crowding, employment status, income proxy |
| Seeman, T.E. et al (2004) | United States | To examine the extent to which a cumulative index of biological dysregulation can explain observed SES differences in mortality riskin a cohort of older adults. | survey | cohort | categorical & dichotomized | no | 10 | None | not significant | no | no | no | educational attainment, allostatic load, age, sex, race, number of chronic conditions |
| Skalická, V. et al (2015) | Norway | Our study examines the extent to which relative educational and income inequalities in mortality might be explained by explanatory risk factors (behavioral, psychosocial, biomedical risk factors and employment) measured at two points in time, as compared to one measurement at baseline. | survey | cohort | categorical & dichotomized | no | 14 | None | mixed | no | yes | yes | education, age, sex, smoking, alcohol, phsyical activity, civil status, feeling lonely, Likert scale, happiness, unemployment status, biomedical factors |
| Sondergaard, G. et al (2012) | Denmark | The aim of the present study was to examine whether indirect selection due to shared genetic makeup and family environment in childhood explained the association between educational level and mortality among full siblings born in Denmark between 1950 and 1979. | census | cohort | categorical | no | 12 | None | mixed | no | no | yes | education, cancer in childhood, psychiatric hospitalization, disabiltiy pension, sex, birth year, birth order |
| Spoerri, A. et al (2014) | Switzerland | Our aim was to use the data of the Swiss National Cohort (SNC), a comprehensive national longitudinal research platform, and evaluate mortality risk and life expectancy in Switzerland based on combined marital (individual and spouse) educational attainment. | census | cohort | categorical | no | 14 | None | positve | no | yes | yes | education, marital status, gender, age, duration of marriage, nationality, type of household, urbanisation, religion, |
| Strand, B. H. et al (2004) | Norway | We investigated the role of classic risk factors and marital status in determining the association between educational level and cardiovascular mortality, with main focus on IHD mortality using individual data collected in 1974–78 in a prospective cardiovascular disease study in the three Norwegian counties. | survey | cohort | dichotomized | no | 12 | Outcome Specific | mixed | no | no | yes | education, marital status, smoking, physical activity, BP, cholesterol, BMI, gender, age |
| Strand, B. H. et al (2005) | Norway | The aim of our study is to determine if the educational gradient in breast cancer mortality persists after adjustment for reproductive history. | census | cohort | categorical | no | 13 | Cancer | mixed | no | no | n/a | education, birth history, age, |
| Sundquist, J. et al (1997) | Sweden | The first aim of this study was to analyse whether self-reported poor health influences total mortality when simultaneously controlling for age, marital status, type of housing tenure, and education. The second aim was to analyse the influence of socioeconomic position, operationalized as the level of educational attainment and type of housing tenure, on total mortality. | survey | cohort | categorical | no | 12 | None | mixed | no | yes | yes | sex, age, marital status, education, type of housing tenure, health status |
| Torssander, J. (2014) | Sweden | This paper studies the associations of children's various socioeconomic resources (education, occupation, and income) and parents' mortality. | census | cohort | categorical | no | 13 | None | significant | no | no | yes | education, occupational class, disposable income combined to give SES position, marital status of parent, SES of parent's partner |
| Turiano, N.A. et al (2014) | United States | We tested whether stronger beliefs of control over one’s life would moderate the effect of education on 14-year mortality risk. | survey | cohort | continuous | no | 11 | None | mixed | no | no | no | age, sex, race, parental education, participant education, control beliefs, self-rated health, etoh use, smoking, depression |
| van Oort, F.V. et al (2004) | Netherlands | The aim of this paper is to simultaneously assess the direct and indirect contributions of material, psychosocial, and behavioural factors to the explanation of educational inequalities in mortality. Furthermore, we aim to point out the relative importance of the three groups of explanatory factors. | survey | cohort | categorical | no | 14 | None | mixed | no | no | no | education, age, smoking, etoh, physical activity |
| van Oort, F.V. et al (2005) | Netherlands | Does the strength of co-occurrence of lifestyle risk factors (smoking, excessive alcohol consumption, and physical inactivity in leisure time) differ by level of education? (2) Do lifestyle risk factors have a synergistic effect on mortality? And (3) does the co-occurrence of lifestyle risk factors have an additional contribution to the explanation of education inequalities in mortality, over and above that of individual risk factors? | survey | cohort | categorical | no | 14 | None | not significant | no | no | no | education, material, behavioral, psychosocial factors |
| Yang, Z.K. et al (2014) | China | We aimed to investigate associations between education level of PD patients’ family members and outcome events, including all-cause and cardiovascular death and first episode peritonitis through a large-scale multi-center retrospective cohort study, which will be helpful for unpacking the black box of the family education-outcome puzzle for PD population. | other | cohort | categorical | no | 11 | Outcome Specific | not significant | no | no | no | age, gender, BMI, primary renal disease, hx of CVD, presence of DM, education of patients and each family member, family income, |
